# Perceptions of, Barriers to, and Facilitators of the Use of AI in Primary Care: Systematic Review of Qualitative Studies

**DOI:** 10.2196/71186

**Published:** 2025-06-25

**Authors:** Héctor Martínez-Martínez, Julia Martínez-Alfonso, Belén Sánchez-Rojo-Huertas, Valeria Reynolds-Cortez, Andrea Turégano-Chumillas, Victoria A Meseguer-Ruiz, Shkelzen Cekrezi, Vicente Martínez-Vizcaíno

**Affiliations:** 1 Health and Social Research Center Universidad de Castilla-La Mancha Cuenca Spain; 2 Integrated Healthcare Service. Primary Care Center Zona 7-Feria Albacete Spain; 3 Daroca Primary Care University Center Madrid Health Service Madrid Spain; 4 Preventive Medicine Hospital Virgen de la Luz Cuenca Spain; 5 Faculty of Health Sciences Universidad Autónoma de Chile Talca Chile

**Keywords:** artificial intelligence, family practice, primary health care, general practice, physicians, systematic review, qualitative research, qualitative evidence synthesis

## Abstract

**Background:**

Artificial intelligence (AI) has the potential to transform primary care by reducing the considerable bureaucratic burden. However, clinicians and patients share concerns regarding data privacy, security, and potential biases in AI algorithms.

**Objective:**

This study aimed to provide an in-depth understanding of primary care professionals’ and patients’ perceptions of, barriers to, and facilitators of the use of AI in primary care.

**Methods:**

We conducted a systematic review of qualitative studies using MEDLINE (via PubMed), Web of Science, and Scopus databases from inception to June 9, 2024. We used the Sample, Phenomenon of Interest, Design, Evaluation, and Research Type (SPIDER) tool to design the systematic search strategy for qualitative studies. Eligible studies included qualitative analyses—based on interviews, focus groups, or similar methods—of perceptions of, barriers to, and facilitators of the use of AI in primary care, involving primary care professionals or patients. Exclusion criteria included studies on clinical decision support systems, reviews, commentaries, editorials, conference abstracts, and non-English or non-Spanish publications. Methodological quality was assessed using the Joanna Briggs Institute checklist. A thematic synthesis approach was used to structure the results, and the Grading of Recommendations Assessment, Development, and Evaluation–Confidence in the Evidence From Reviews of Qualitative Research (GRADE-CERQual) tool was used to assess the confidence in each finding.

**Results:**

We analyzed 316 participants, including primary care physicians, patients, and other health care professionals, from 13 studies across 6 countries selected from 942 screened records. We identified four analytical themes using thematic synthesis: (1) change in the physician-patient relationship, highlighting concerns about loss of empathy, connection, and trust; (2) AI as a partner for efficient time and information management, including its potential to improve workflow and decision-making, alongside skepticism about increased workload; (3) data as the cornerstone of AI development, reflecting concerns about data privacy, quality, bias, and corporate responsibility; and (4) barriers to and facilitators of AI in primary care, emphasizing equity, accessibility, and stakeholder co-design. The GRADE-CERQual assessment provided high confidence in all themes except theme 4, which was rated as moderate confidence.

**Conclusions:**

This meta-synthesis includes the perspectives of primary care physicians and patients, but further research is needed on the perspectives of other professionals. Moreover, there was heterogeneity in methods and sampling strategies. This first systematic review synthesizing qualitative evidence on AI perceptions in primary care provides a comprehensive understanding of related barriers and facilitators. The key themes identified suggest that AI may help address workload, decision-making, and data management, improving health care efficiency while ensuring ethical, patient-centered care.

**Trial Registration:**

PROSPERO CRD42024560048; https://www.crd.york.ac.uk/PROSPERO/view/CRD42024560048

## Introduction

### Background

Artificial intelligence (AI) has led to a paradigm shift in the field of health care. Advances in machine learning, natural language processing, and computer vision have substantially improved several areas of medical practice, such as diagnostic accuracy, image interpretation, and personalized medicine. All these tools use data from various sources, including demographic data, medical records, physical and laboratory examinations, and images [1]. Large volumes of electronic health records are one of the key drivers of AI development in medicine. However, this efficient use of clinical data is both the main technological advance and its primary constraint because the integration of AI into clinical practice raises ethical concerns, such as data privacy, transparency of AI decision-making, and bias in predictive algorithms [2], which often lead to inequities in care [3].

Primary care is the heart of the quality of health systems [4], but if burnout and the shortage of primary care clinicians continue to increase, the entire health care system could deteriorate to the point where it becomes intolerable for both the general population and medical professionals [5]. It has been estimated that providing good quality care to 2500 primary care patients requires 27 hours per day [6]. Surprisingly, electronic health records, which appeared to facilitate the clinical management of patients and improve the efficiency of family physicians, have become the main problem, as primary care clinicians spend much of their time navigating and clicking through digital interfaces [7].

AI has the potential to improve and transform primary care clinical practice by reducing the considerable bureaucratic burden that has significantly permeated it [8]. Three groups of tasks have been identified where AI could benefit clinical work and improve the quality of care provided: inbox and documentation management, time management between visits, and decision support for diagnosis and treatment [9]. However, the integration of AI tools in primary care also presents unique challenges, as the management of patients in primary care is more complex than in any other setting, requiring more nuanced clinical judgment and a deep understanding of the context in which the patient lives [4]. It is precisely these unique aspects of primary care where AI systems face their greatest limitations [10], highlighting the need for participatory reflection on regulatory and ethical perspectives—reflection that includes input from all stakeholders across the health care ecosystem [11].

Several qualitative studies have been conducted to gain insight into the public perceptions of AI in medicine [12,13]. These studies have identified recurring themes, including the need for transparent ethical and legal frameworks, the importance of developing trust in AI systems, and the critical role of education and training in facilitating AI integration. Furthermore, various factors that influence health care professionals’ attitudes toward AI have also been identified. Consequently, it is imperative to comprehensively address these concerns to ensure the successful and ethical integration of AI into medical practice; however, no study has yet synthesized the available evidence on these issues in primary care.

### Objectives

Therefore, this study aimed to provide a synthesis of the qualitative scientific evidence that facilitates an in-depth understanding of primary care professionals’ and patients’ perceptions, barriers, and facilitators regarding the use of AI in primary care. Our synthesis may help to implement AI while considering the perspectives of patients and primary care professionals to ensure an effective, ethical, and patient-centered implementation of AI in primary care settings.

## Methods

This review was conducted according to the Cochrane Qualitative and Implementation Methods Group [14], registered in the PROSPERO database (CRD42024560048), and reported according to the Enhanced Transparency in Reporting the Synthesis of Qualitative Research (ENTREQ) statement [15] (Multimedia Appendix 1) and the Evidence-Based Measures of Empowerment for Research on Gender Equality (eMERGe) reporting guidelines for meta-ethnography studies [16].

### Search Strategy and Selection Criteria

A comprehensive literature search was undertaken in the MEDLINE (via PubMed), Web of Science, and Scopus databases from inception to June 9, 2024. We used the Sample, Phenomenon of Interest, Design, Evaluation, and Research Type (SPIDER) tool as a systematic search strategy for qualitative studies [17]. The search strategy combined the following terms: “artificial intelligence,” “primary care,” “family medicine,” and “perception” (Multimedia Appendix 2). The search strategy was modified according to each specific database requirement. In addition, the reference lists of selected studies were screened.

To be included in the review, studies had to report a qualitative analysis of the perceptions, barriers, or facilitators associated with the use of AI in primary care. Qualitative methods, such as semistructured interviews, focus groups, or alternative approaches, were required for data collection. In addition, for studies using a mixed methods approach, where quantitative and qualitative data are presented separately, only the qualitative data subject to analysis were included.

Participants included all primary care professionals and patients. Only studies published in English or Spanish were included. Articles exclusively focused on clinical decision support systems were excluded from the analysis. Conference proceedings and abstracts, reviews, editorials, and commentaries were also excluded.

### Research Screening and Data Extraction

The search results were imported into the Rayyan citation manager [18], and duplicates were removed. Two authors (HM-M and JM-A) independently screened studies against the predefined inclusion and exclusion criteria. Studies potentially meeting the criteria were subjected to full-text screening for the final decision on inclusion in our review. Discrepancies at any stage were resolved through discussion. If consensus was not reached, a third reviewer (VM-V) made the final decision. A full description of the procedures used to screen and extract data from the literature is provided in Multimedia Appendix 2.

### Quality Assessment

The methodological quality and risk of bias of the included studies were assessed using the Joanna Briggs Institute Critical Appraisal Checklist for Qualitative Research [19], which consists of 10 statements addressing the possibility of bias in study design, conduct, and analysis (Multimedia Appendix 3 [20-32]). Two authors (HM-M and JM-A) independently applied the checklist, and disagreements were resolved by consensus.

### Data Extraction and Synthesis

Two independent reviewers (HM-M and BS-R-H) extracted data using a standardized form. Data obtained included the following: authors, year of publication, country, data collection methods, analytical approach, data analysis software, participant characteristics (sample size, sex, age, and profession), place, and method of recruitment.

HM-M conducted a critical analysis of the included articles, identifying and extracting the key themes, subthemes, and quotations. The identified themes were subsequently categorized to develop concepts and construct meaning for discussion.

Using the thematic synthesis method outlined by Thomas and Harden [33], 2 authors (HM-M and JM-A) conducted line-by-line coding of texts and quotations from the results sections of the included studies. They independently grouped similar codes to generate descriptive themes, reached a consensus through discussion, and consulted a third author (VM-V) when necessary. This phase was conducted from a descriptive and inductive stance, aiming to remain close to the participants’ discourses. In the final stage, HM-M interpreted the descriptive themes to identify overarching insights across the studies, adopting a more interpretative position to construct analytical themes that extended beyond the findings of the individual studies.

### Confidence in the Evidence Obtained

The Grading of Recommendations Assessment, Development, and Evaluation–Confidence in the Evidence From Reviews of Qualitative Research (GRADE-CERQual) approach was used to assess confidence in each synthesized finding, categorizing it as high, moderate, low, or very low. This assessment considers 4 dimensions of the evidence: methodological limitations, coherence, data adequacy, and relevance. Each dimension was independently evaluated by 2 reviewers (HM-M and JM-A), and findings were initially rated as “high confidence,” with potential downgrades based on the severity and number of concerns identified. Disagreements were resolved through discussion with a third author (VM-V). The final confidence level reflects the cumulative assessment of these components [34-39].

## Results

### Overview

**Figure 1:**
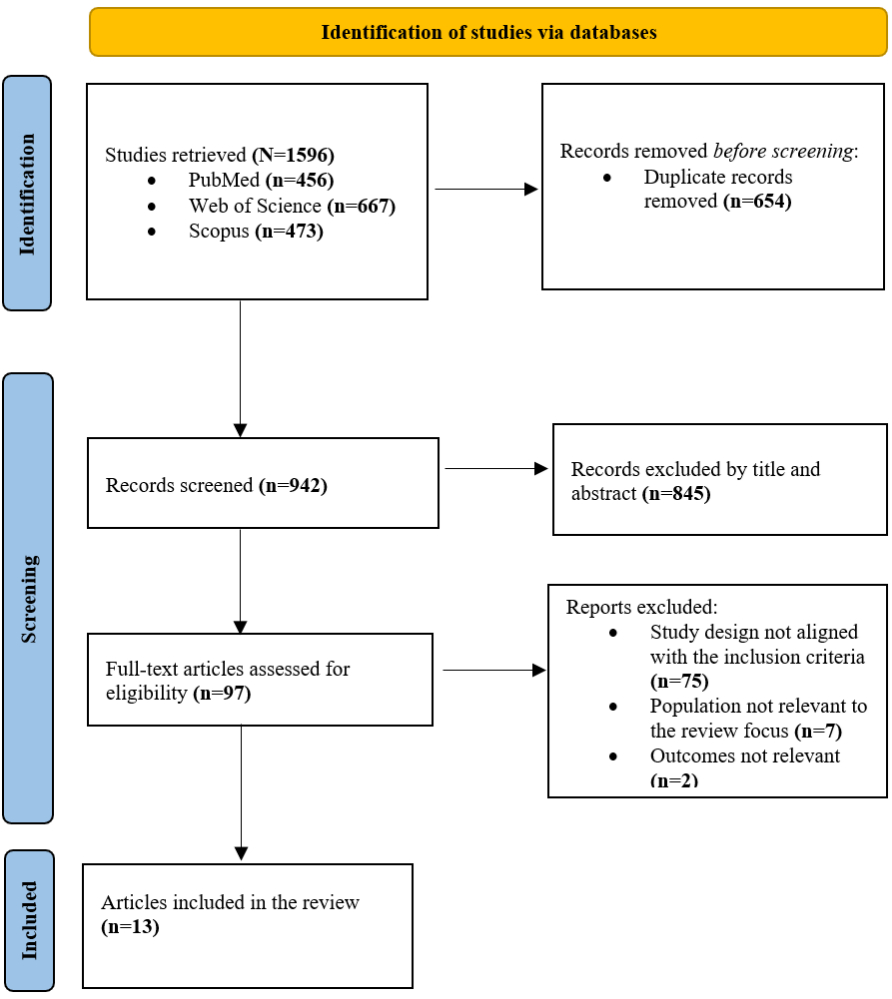
PRISMA (Preferred Reporting Items for Systematic Reviews and Meta-Analyses) 2020 flow diagram illustrating the progression of studies through the review process.

After removing duplicates, 942 studies were screened by title and abstract, and 97 articles were included for full-text review to assess eligibility. Finally, 13 articles from 11 studies were included (Figure 1). Our final analysis includes 11 unique studies, which are reported across 13 articles. Some publications originate from the same primary study.

Of the 13 articles, 3 (23%) were conducted in Canada [20-23], 2 (15%) in the United States [24-26], 2 (15%) in Australia [27,28], 2 (15%) in Germany [29,30], 1 (8%) in the United Kingdom [31], and 1 (8%) in Denmark [32]. Most studies (12/13, 92%) used primarily qualitative methodologies, and of the 13 studies, 1 (8%) used a mixed methods approach [24]. Data collection methods included individual interviews in 54% (7/13) of the studies, focus groups in 8% (1/13) of the studies [25,26], workshop activities in 8% (1/13) of the studies [27], deliberative dialogues in 8% (1/13) of the studies [20,21], and surveys with open-ended questions in 8% (1/13) of the studies [31] (Table 1).

**Table 1:** Description of the included studies.

Study	Country	Methodology of data collection	Methodology of analysis	Characteristics of the participants	Place and recruitment method
Darcel et al [20], 2023	Canada	Semistructured interview format with an interview guide via the Zoom conferencing system	Data analysis using Microsoft Excel and Word	N=48. Patients (n=22), primary care providers (n=21), and health system leaders (n=5) Age (years): 23-73 Gender—men: n=21, women: n=21, and nonbinary: n=1	Selection by convenience sampling from various online advertisement platforms and email distribution lists
Upshaw et al [21], 2022	Canada	Semistructured interview format with an interview guide via the Zoom conferencing system	Data analysis using Microsoft Excel and Word	N=48. Patients (n=22), primary care providers (n=21), and health system leaders (n=5) Age (years): 23-73 Gender—men: n=21, women: n=21, and nonbinary: n=1	Selection by convenience sampling from various web-based advertisement platforms and email distribution lists
Terry et al [22], 2022	Canada	Interviews via the Zoom conferencing system or telephone	Thematic analysis using NVivo software	N=14. Decision makers (n=4), decision maker or researchers (n=2), primary health care practitioner or researchers (n=5), and researchers (n=3)	Selection by convenience sampling generated through the investigator team member’s existing networks and a search of relevant organizational websites and publications
Nash et al [23], 2023	Canada	Semistructured interview format with an interview guide in person, with 2 by videoconference and 1 by telephone	Thematic analysis using NVivo 11 software	N=26. Health care providers (n=10), executive directors (n=8), and data support staff (n=8)	Selection by convenience sampling from 73 community health centers
Allen et al [24], 2024	United States	Semistructured interview format with an interview guide via the Zoom conferencing system	Thematic analysis using Quirkos software	Primary care physicians (N=15) Age (years): 25-64	Participants were affiliated with an academic medical center
Richardson et al [25], 2022	United States	Focus groups followed a semistructured, case-based format	Data analysis using NVivo 11 software	Patients. (N= 87; 15 focus groups) Age (years): 18-91 Gender—men: n=44 and women: n=43	Convenience sampling selection of patients who visited a Mayo Clinic primary care facility in Minnesota or Wisconsin
Richardson et al [26], 2021	United States	Focus groups followed a semistructured, case-based format	Data analysis using NVivo 11 software	Patients (N=87; 15 focus groups) Age (years): 18-91 Gender—men: n=44 and women: n=43	Convenience sampling selection of patients who visited a Mayo Clinic primary care facility in Minnesota or Wisconsin
Kocaballi et al [27], 2020	Australia	Semistructured interviews format with an interview guide via the Zoom conferencing system	Thematic analysis using NVivo 12 software	Primary care physicians (N=16) Gender—men: n=10 and women: n=6	Selection by convenience sampling from website information, social media, and primary health care networks’ email newsletters
Fraile Navarro et al [28], 2023	Australia	Semistructured interviews format with an interview guide via the Zoom conferencing system and 1 interview conducted by a person	Data analysis using NVivo software	Primary care physicians (N=10) Gender—men: n=7 and women: n=3	Recruitment through invitation leaflets, sampling, and reaching out to the local health district
Buck et al [29], 2022	Germany	Semistructured interviews format with an interview guide in person and via phone	Thematic analysis using MAXQDA 2020	Primary care physician (N=18) Age (years): 34-70 Gender—men: n=9 and women: n=9	Selection by convenience sampling within the geographic reach of the research team
Kamradt et al [30], 2022	Germany	Semistructured interviews format with an interview guide via telephone	Data analysis using MAXQDA Plus 2018 software	Primary care physicians (n=6) and hospital-based physicians (n=2; not included) Age (years): 30-74 Gender—men: n=7 and women: n=1	Selection by convenience sampling using known contacts in academic teaching practices and personal contacts in hospital departments in Baden-Wuerttemberg
Blease et al [31], 2019	United Kingdom	Anonymous nationwide web-based survey of UK primary care physicians. This survey instrument included a single open-ended question	A full thematic analysis was not applicable. Responses were collated and imported into QCAmap for analysis	Primary care physicians (N=66) Age (years): >45 (n=55 participants) Gender—men: n=38 and women: n=28	Participants were randomly sampled from the membership of the clinician marketing service Doctors
Mikkelsen et al [32], 2023	Denmark	Interviews were conducted with the vignette method	Data analysis using NVivo 13 software	Patients (N=10) Age (years): 25-74 Gender—men: n=4 and women: n=6	All the patients were recruited from the North Denmark Region through different Facebook groups

### Characteristics of Participants

Of the 11 studies, 8 (73%) reported participants’ gender [20,21,25-32], with 140 men, 117 women, and 1 nonbinary participant. In total, 55% (6/11) of the studies exclusively involved primary care physicians [24,27-31], 18% (2/11) involved patients [25,26,32], and 27% (3/11) included participants from multiple professions within the primary care setting [20-23]. Our analysis included 316 participants: 131 (41.5%) primary care physicians, 119 (37.7%) patients, and 66 (20.9%) other health care professionals from diverse primary care settings. This diverse sample provided comprehensive insights into various health care perspectives.

### Thematic Synthesis

**Figure 2:**
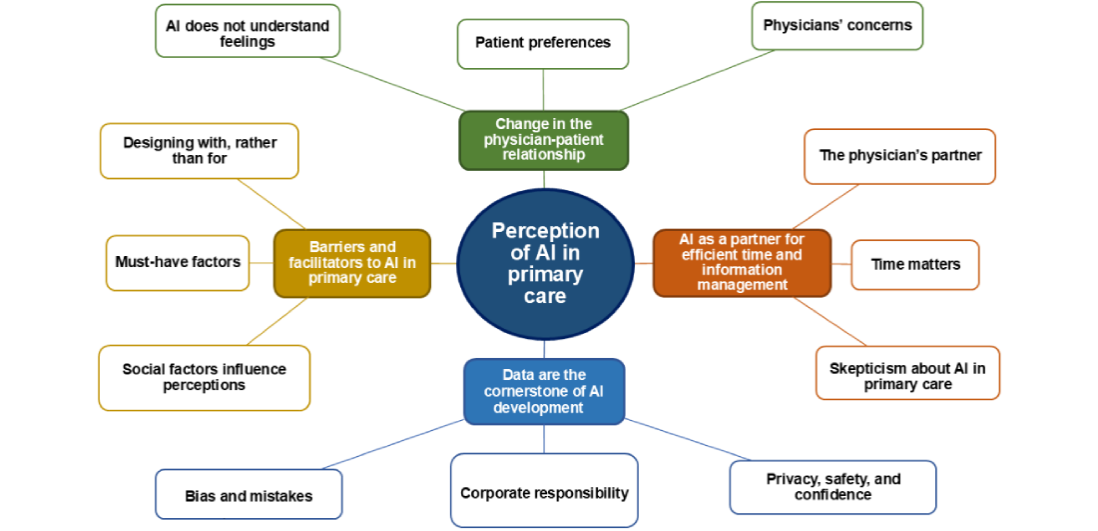
Graphical representation of the themes and subthemes. AI: artificial intelligence.

Thematic synthesis revealed 4 main themes, each comprising 3 subthemes and each supported by relevant quotations (Figure 2). All quotations, organized according to themes and subthemes, are presented in Multimedia Appendix 4 [20-32]. In addition, a summary of selected key quotations illustrating each theme and subtheme is provided in Table 2.

**Table 2:** Thematic framework with representative participant quotations.

Themes, subthemes, and participant quotations				References
**Theme 1: change in the physician-patient relationship**				
	**AI^a^ does not understand feelings**			
		“I think the point of the doctor is to give suffering meaning. It’s to provide a steady hand, I think. It’s to support people through issues...a computer can support you, but it doesn’t have any meaning because there’s no emotional risk from the computer because the computer does not know what it means to live or die.” [Physician]	[27]	
		“I’m much more comfortable with AI as an assistive tool, rather than making decisions on its own.” [Health care professional]	[22]	
		“Technology cannot replace doctors. There is definitely a 6th sense.” [Physician]	[30]	
	**Patient preferences**			
		“I want my doctor to be present with me in a conversation and not staring at the computer and looking at all the tools or boxes that they need to check off.” [Patient]	[20]	
		“They [patients] can do it [have the consultation] at home. You feel like rubbish in the morning, do you really want to have to call the GP, get an appointment, sit in a waiting room for an hour with other coughs and colds...think of all that disease that’s spread in a waiting room.” [Physician]	[27]	
		“You know what, that’s just like for me, I have multiple sclerosis, but I also have fibromyalgia, so the thing is when I hurt, I can’t tell you why it hurts, you know what I’m saying? I can’t tell you, oh my fibromyalgia’s kicking up or I’m having a MS attack or whatever. I can’t tell you the difference, and if I can’t tell you the difference, I know AI cannot tell me the difference.” [Patient]	[25]	
	**Physicians’ concerns**			
		“Patients may end up feeling that, you know, if the AI can tell me that then why did I bother to come to you?” [Physician]	[24]	
		“I think eventually it [AI] will take all the jobs. We’re going to be the last group anyway to use this.” [Physician]	[27]	
		“My greatest fear is that you lose your critical thinking because something’s going to come up on a screen and tell you what to do.” [Health care professional]	[23]	
**Theme 2: AI as a partner for efficient time and information management**				
	**The physician’s partner**			
		“I think the goal of medicine is to either heal you or to live a more fuller, richer life, and I think if AI with all its knowledge can help do that, if it can help me get better, it’s like that extra little thing that...you know, that extra training that’ll make me a better runner, that extra math class that’ll help make me smarter in math. It’s just that little extra that maybe will achieve that goal of, like I said, leading healthier lives.” [Patient]	[25]	
		“It will make life easier, and it will integrate and maybe improve care for the patient, that is what I’m thinking.” [Physician]	[28]	
		“You just tell the computer what [you] want to check and then the computer will do the job and generate the report.” [Physician]	[27]	
	**Time matters**			
		“First of all, [AI] should be fast. There is always time pressure.” [Physician]	[27]	
		“As a patient, I don’t want my doctor spending his time facing the computer. I want him facing me...So in terms of looking at all of those admin tasks that are taking away from the patient care, I think AI has the potential to free up that time so that I have more face time with my doctor.” [Patient]	[21]	
		“I think that doctors are often overwhelmed and overworked and if AI can be used to help with that, I’m all for that, so that they can be more efficient and more effective in their work. [Patient]”	[21]	
	**Skepticism about AI in primary care**			
		“We are going to add two extra patients per session because now we have help there. So unfortunately, sometimes more help is used in a negative way.” [Physician]	[24]	
		“Is it really the physicians that should deal with this in the first place?” [Physician]	[24]	
		“[I]n routine cases, [AI] would not be a time saver for me.” [Physician]	[29]	
**Theme 3:** **data as the cornerstone of AI development**				
	**Privacy, safety, and confidence**			
		“Of course, it is also important to me that there is corresponding data security. I do not want the patients and us to be completely transparent. That is certainly not in the overall interest.” [Physician]	[29]	
		“I was just gonna say another concern that I think I would have, just because of the way our world is evolving and revolving, is can that artificial intelligence be hacked? Who can control that?...I don’t know. Because any time you have a computerized program, I don’t care what anybody says, it can and it will get hacked because there’s always somebody that’s out there just to do evil rather than good.” [Patient]	[26]	
		“It’s always the problem of hacking, and somebody can know what exactly you do and can track the patient record.” [Physician]	[27]	
	**Corporate responsibility**			
		“The problem is that large companies use AI to gain access to lucrative patients and to control them via AI.” [Physician]	[29]	
		“[T]he business model behind these technologies is where I think the most important ethics conversation is to be had at the moment.” [Health care professional]	[22]	
		“The other part is [what is] behind the—maybe the dirty side of AI, which is the monetization of big data.” [Health care professional]	[22]	
	**Bias and mistakes**			
		“The thing I’m apprehensive about is, how are we teaching AI these things because some of those biases could leak in.” [Physician]	[24]	
		“AI is not a living creature and depending on what you feed it with, it can learn different things, so it is important to be critical.” [Patient]	[32]	
		“We want to make sure that the tools we use don’t create new problems...they are an opportunity to address some of the biases that already exist in our system...and that these tools are probably only as good as the data we provide them.” [Physician]	[26]	
**Theme 4: barriers and facilitators regarding AI in primary care**				
	**Social factors influence perceptions**			
		“So it sounds expensive, and health care is already fairly expensive. To go on his note, a lot of times you can get something that works just as well for a lot less or you could get something super fancy, that makes you think, hey I got this big fancy thing, but it really doesn’t do any better than the original cheaper version.” [Patient]	[26]	
		“I am convinced it needs much work because there is certainly much resistance, which clearly depends on age.” [Physician]	[29]	
		“In essence, we’re providing a bunch of free care, which, you know, is not sustainable.” [Physician]	[24]	
	**Must-have factors**			
		“I think it all comes back to choice, though, I think everybody’s getting the mentality that, and maybe I’m wrong, but that an AI is being pushed, but at the end of the day, our choice is still our choice, and it’s not being taken away.” [Patient]	[26]	
		“At this point, I want to be able to get a logical explanation.” [Physician]	[24]	
		“As long as I could give the final yes or no.” [Physician]	[28]	
	**Designing with, rather than for**			
		“Instead of just letting the cat out of the bag and seeing what happens, you want to make sure that everyone that is going to be interacting with it has accurate expectations and has been educated on what role this is supposed to play.” [Health care professional]	[24]	
		“[T]hat co-design piece of having the end users—so most likely nurses, doctors, nurse practitioners, anyone who’s going to be using the technology really needs to be involved in the development and co-design of the technology from the beginning...right now, with other types of technology, it will be this tech company that’s developing this great system and they only consult the end users when it’s finished and then it’s almost too late to kind of incorporate things that really should’ve been included from the beginning. So that is something that would be really important to emphasise and could help with a successful implementation of any AI technology as well.” [Health care professional]	[22]	

^a^AI: artificial intelligence.

#### Theme 1: Change in the Physician-Patient Relationship

##### AI Does Not Understand Feelings

Participants across the studies consistently highlighted the limitations of AI in developing crucial humanlike competencies for clinical practice [21-24,27-29,31,32]. They maintained that AI cannot fully replace human clinicians, as only humans can truly demonstrate empathy and possess the unique capacity to understand patients’ beliefs, fears, and individual circumstances. When asked to share their views on responsibility for decision-making in clinical practice with AI, they expressed skepticism about this new tool [29]:

Experience can hardly be replaced by AI. Experience and intuition. And empathy. This is just how I treat people, to get something out of them. So, this is something that defines a good physician and cannot be replaced by AI. Empathy.

Physician

##### Patient Preferences

Most patients emphasized the importance of the physician’s role in health care provision [20,21,25-27,29,31,32]. However, the potential transformation of the physician-patient relationship by technological advancement raised doubts among many respondents [29,32]:

My experience every day with patients is that they want to be touched, and they want to look you in the eyes.

Physician

A person does not have a trustful relationship with a machine.

Patient

##### Physicians’ Concerns

Despite the potential benefits in primary care, some physicians worried that AI could undermine the value of their recommendations during clinical interviews, reducing their function to that of a mere data collector on a computer [20,22-24,27,29]. Some participants expressed concerns about the possibility of job loss if patient trust were to diminish [27]:

If they [patients] think that we're just getting suggestions from a computer, then maybe they can just get suggestions from a computer. I think it becomes more difficult to convince them that our recommendations are more valuable than what they can pick up on the internet.

Physician

#### Theme 2: AI as a Partner for Efficient Time and Information Management

##### Overview

Participants across all the studies anticipated substantial changes in the practice of medicine in the near future. They identified the integration of AI as a key strategy to address the growing demand for health care services, which often exceeds the current system’s capacity [21,32]:

It is a radical change compared to how health is being practiced now.

Physician

I think that doctors are often overwhelmed and overworked and if AI can be used to help with that, I’m all for that, so that they can be more efficient and more effective in their work.

Patient

##### The Physician’s Partner

AI’s ability to process and analyze large datasets would facilitate efficient patient data organization, enhancing data accessibility and use for health care professionals. [20-23,25,27-29,31,32] Participants also noted that AI integration could improve diagnostic procedures by offering solutions that would otherwise require a clinician considerable time to identify. Furthermore, participants believed that AI could reduce diagnostic errors and improve clinical decision-making. Among the specific applications mentioned, participants highlighted the potential of AI to improve the accuracy of medical diagnoses, streamline the preparation of clinical documentation, and support triage processes in primary care. It is widely acknowledged that the goal of these developments is to enhance the quality of life for both clinicians and patients [25]:

I think it’s good to have the more input. The more input that comes into here, the better I think it’s going to be able to respond to a situation. We definitely all agree, I think, in this room that the more information that is collected, the better off all people are gonna be...

Patient

##### Time Matters

Many of the tools envisioned by respondents focus on time management as their primary objective, eliminating repetitive and bureaucratic tasks [20,21,24,25,27-30]. As a result, some participants anticipated a reduction in the burden of care and an increase in patient interaction time [28]:

Yes, just taking my hands off the computer, getting my eyes off the screen, so that I can be spending time with the patient. And also saving me the documentation time, because you can either spend more time with the patient or see more patients.

Physician

##### Skepticism About AI in Primary Care

Not all participants were optimistic about the potential of AI in medicine [20,22,24,27-29,31]. There was a sense that the use of AI-enabled systems may not work as expected and may even increase the burden of care rather than reduce it. It was noted that the implementation of these technologies might require additional effort in terms of training, monitoring, and management of complex systems. The anticipated benefits might be outweighed, leading to more challenges than solutions in the context of clinical care [24]:

It’s like having a student with me all the time, where I’ve got to just double check everything.

Physician

#### Theme 3: Data as the Cornerstone of AI Development

##### Privacy, Safety, and Confidence

Participants emphasized that patient data play a crucial role in the development of AI in health care [20-24,26-32]. Some noted that meaningful progress in AI technologies would not be possible without reliable and secure access to such information [30]:

This is indispensable, so if you want to develop artificial intelligence in the medical context, you cannot do it without patient data.

Physician

However, given the sensitivity of the medical information involved, some participants expressed reservations about the potential violation of their right to privacy. Ensuring anonymity and demanding transparency regarding the identity of those responsible for managing the available information were considered essential [30]:

[I]t must be ensured that data is deidentified...Overall, the process must be transparent, you must always be able to understand what is done with the data and how it is processed. Exactly...who processes the data, what research projects are being carried out...

Physician

The integration of AI raised apprehensions among physicians regarding patient safety and the potential impact of continuous data monitoring on their own safety and legal responsibilities. Physicians were worried that constant monitoring may compromise their professional autonomy, leading to a sense of being observed at every step. This may restrict their ability to make independent clinical decisions and create an environment of distrust and pressure that could affect both their medical practice and personal well-being [28]:

If everything was completely recorded and stored...the entire conversation, if that was recorded and stored, and the doctor missed something, then that could be used against the doctor. That the patient mentioned it, but it wasn’t listed or written, or a doctor accidentally missed that detail. Then that could be a problem.

Physician

##### Corporate Responsibility

Many stakeholders identified the role of business in AI development as a key area of concern [20,22,23,25,29]. There was a perception that the existing legal framework may not adequately protect against the potential misuse of data by certain organizations [22]:

[What] we need to add is a way to make sure that personal health information is protected and that we don't step outside social licence.

Health care professional

Participants noted that current AI applications are being developed by private, profit-driven entities, which may result in a lack of transparency and a prioritization of business interests over patient welfare. Some even suggested that companies might influence the algorithms that process data to serve their own interests [25]:

Like who decides what’s good or bad? It’s relative depending on whatever company wants to make a bunch of money off their data. That’s what I’m the most nervous is about the corporate side of it. Who is regulating it? Who is saying this algorithm is good to go? There’s no...there isn’t that yet.

Patient

##### Bias and Mistakes

Participants identified the introduction of erroneous data into AI-based systems as a potential source of bias [20-26,28,29,32]. In addition, preexisting biases may be reinforced during the development of the algorithms or through systematic errors in the learning process. For some individuals, these factors could influence the decision-making processes of primary care physicians, potentially leading them to overlook more optimal alternatives due to their reliance on AI recommendations [23]:

I think the risks would be if somebody inputted something wrong and it then prompting you to do something. Like the data at the end is only as good as how you input the data at the beginning.

Health care professional

Furthermore, participants identified a potential issue with the representativeness of the data used in AI tool training, underscoring the risk that certain social groups may be disadvantaged. The discussion often focused on demographic variables, such as race and gender [27]:

A lot of the input data coming so far is from white males—so a lot of the algorithms and all of the learning at the moment is largely based on white male thinking...so there are inherent biases already within their programs in the way that [data is being collected]—even the idea of drawing conclusions about the diagnosis.

Physician

#### Theme 4: Barriers and Facilitators Regarding AI in Primary Care

##### Social Factors Influence Perceptions

Individuals’ perceptions of AI-related changes in their environment are influenced by their social context, including factors such as age and socioeconomic status (SES) [20,24-27,29-32]. Concerns have been raised that advances in AI would mainly benefit those who could afford them, potentially excluding populations considered economically disadvantaged from receiving optimal medical care. In addition, the high cost of these technologies could further strain health care systems, potentially prioritizing cost-efficiency over medical necessity [24]:

The people who will get it are the people who can pay for the compute. And so that’s my biggest fear is that we will leave out the poorest people from getting the best care.

Physician

##### Must-Have Factors

A variety of perspectives were presented when discussing the characteristics that AI should have in the context of health care [20,21,23,24,26-31]. A common theme emerged, namely, the importance of AI being able to adapt to the preferences and styles of health care professionals, thereby supporting their consultation and decision-making processes [27]:

We need to develop machine learning that has the capability...to accommodate professional preferences and styles...It’s adaptive and it supports you based on your particular consultation and decision-making processes.

Physician

Furthermore, participants emphasized the need for AI to be accessible, reliable, and capable of optimizing workflow without increasing the overall workload. They also reiterated the importance of rigorous testing to ensure the effectiveness of the system and to safeguard that both medical professionals and patients maintain control over the use of the data [26]:

So when this intelligence is built we have to test it, right? We have to test it to make sure that it’s helping correctly, and that to me represents a big challenge and one we don’t wanna jump into and see what happens. We’ve gotta be very careful there.

Patient

##### Designing With, Rather Than For

Studies have shown that the successful implementation of AI in primary care settings requires the active involvement of a range of stakeholders [20,22,24]. The concept of co-design is crucial; health care professionals, including physicians, nurses, patients, and other end users, must be actively involved from the earliest stages of AI tool development. The involvement of these actors in the design process, rather than simply passively receiving the resulting technology, will ensure that the resulting tools meet the actual needs of the clinical environment, thereby facilitating a more efficient and effective adoption process. This collaborative approach facilitates not only the incorporation of feedback from health care professionals but also transparency regarding the identity of the designers of the tools and the data used to train the models. Such transparency is seen as essential to building confidence [22]:

I think that they [patients and practitioners] [need to] know what goes into the black box. I’d want to make [sure] that it was actually explained and articulated how the end result came about. I just wouldn’t want any assumptions to be made. Sometimes at the system level decisions are made that may not take into consideration all the different aspects. I don’t think that would happen but having all of the different pieces put together is important; the contextual pieces.

Health care professional

#### Assessment of Confidence

The GRADE-CERQual assessment provided confidence ratings for the 4 themes outlined in the results, as summarized in Table 3.

**Table 3:** Summary of the qualitative findings with the Grading of Recommendations Assessment, Development, and Evaluation–Confidence in the Evidence From Reviews of Qualitative Research (GRADE-CERQual) assessments.

Themes and summarized review findings			GRADE-CERQual assessment of confidence		Explanation of the GRADE-CERQual assessment		References
**Change in the physician-patient relationship**							
	AI^a^ lacks the human-specific qualities essential for patient care, such as empathy and understanding of feelings, raising concerns about its impact on the physician-patient relationship and trust. Physicians also fear being reduced to data collectors and patients devaluing their clinical advice, potentially affecting job security.	High confidence		Minor concerns regarding methodological limitations (1 study used open-ended survey questions [31]). This paper does not change the common ideas of the final conclusions based on the quotations of the participants. No or very minor concerns regarding coherence, no or very minor concerns regarding adequacy, and no or very minor concerns regarding relevance.		[20-29,31,32]	
**AI as a partner for efficient time and information management**							
	The integration of AI in health care is expected to transform the field by improving data processing, diagnostic accuracy, and clinical decision-making. While some anticipate reduced workloads and increased patient interaction for caregivers, others worry that AI systems may not perform as expected, potentially increasing the overall burden of care.	High confidence		Minor concerns regarding methodological limitations (1 study used open-ended survey questions [31]). This paper does not change the common ideas of the final conclusions based on the quotations of the participants. No or very minor concerns regarding coherence, no or very minor concerns regarding adequacy, and no or very minor concerns regarding relevance.		[20-25,27-32]	
**Data as the cornerstone of AI development**							
	Participants raised concerns about the ethical and practical implications of integrating AI into health care, highlighting issues such as privacy violations, patient safety, data transparency, and biases in AI systems. There were also worries about the impact on medical autonomy, with constant data monitoring potentially limiting clinical decision-making.	High confidence		Minor concerns regarding methodological limitations (1 study used open-ended survey questions [31]). This paper does not change the common ideas of the final conclusions based on the quotations of the participants. No or very minor concerns regarding coherence, no or very minor concerns regarding adequacy, and no or very minor concerns regarding relevance.		[20-32]	
**Barriers and facilitators regarding AI in primary care**							
	The social context influences how patients view their role in a technology-driven primary care system. Successful AI implementation in health care requires adaptability to professional preferences and a co-design approach involving health care professionals and patients to ensure that tools meet clinical needs, promote transparency, and ease adoption.	Moderate confidence		Minor concerns regarding methodological limitations (1 study used open-ended survey questions [31]). This paper does not change the common ideas of the final conclusions based on the quotations of the participants. Moderate concerns regarding coherence (the participants’ ideas were subjected to an interpretative process), no or very minor concerns regarding adequacy, and no or very minor concerns regarding relevance.		[20-32]	

^a^AI: artificial intelligence.

The synthesis of findings in all themes was rated as high confidence, except for theme 4, “barriers and facilitators regarding AI in primary care,” which was rated as moderate confidence (detailed information on all items is provided in Multimedia Appendix 5 [20-32]).

## Discussion

### Overview

To the best of our knowledge, this is the first study to synthesize qualitative scientific evidence to support an in-depth understanding of primary care professionals’ and patients’ perceptions of, barriers to, and facilitators of the use of AI in primary care. Our analysis of the included studies represented the voices of key primary care stakeholders, such as leaders, physicians, and patients, and revealed 4 key themes: the change in the physician-patient relationship, AI as a partner for efficient time and information management, data as the cornerstone of AI development, and barriers and facilitators regarding AI in primary care.

### Change in the Physician-Patient Relationship

The patient-physician relationship is a dynamic entity that has evolved over time to reach the current paradigm of patient-centered medicine [40]. While the importance of the physician’s role in the clinical care process is widely recognized and generally accepted, the emergence of AI in primary care clinical settings has introduced new uncertainties and doubts, with some participants seeing it as a potential threat to the physician-patient relationship. A major concern is the erosion of trust between patients and physicians due to increasing reliance on AI. In today’s information-rich environment, medical professionals worry that patients may have unrealistic expectations, leading to a shift in decision-making authority to AI. If patients perceive AI as more reliable and accurate, they will no longer place their trust in their primary care physician to solve their health problems.

This prompts the interviewees to consider the evolving function of primary care physicians. The delegation of greater authority to AI tools could result in the physician becoming a mere collector of information, potentially compromising their status within the medical profession. Patients had dissenting opinions regarding the extent to which AI should be involved in their overall health care journey. Some respondents emphasized the importance of primary care physicians as a source of expertise in solving health problems and as a provider of care and empathy. Conversely, other interviewees were more inclined to cede authority to AI, viewing this as an opportunity to enhance their relationship with medicine by accessing broader expertise beyond the scope of their primary care physician.

In recent years, the development of AI capable of demonstrating empathy has opened up new possibilities, with promising results observed in the management of mental health [41,42]. However, as this technology continues to evolve, it necessitates reflection on the future of primary care and its role in shaping the landscape of medicine in the coming decades.

### AI as a Partner for Efficient Time and Information Management

A total of 3 positive suggestions were made by the participants that may help in the day-to-day clinical work: the potential for AI to improve the accuracy of medical diagnoses; the optimization of information management processes, including the preparation of clinical reports; and the introduction of triage tools. These potential applications are seen by primary care physicians as a means to enhance diagnosis accuracy and, overall, the clinical management of patients. As described in the Results section, such tools were also perceived to reduce administrative burden, enabling clinicians to dedicate more time to the relational and humanitarian aspects of care. In parallel, patients expressed a desire for more personalized and attentive interactions with their health care providers, believing that the integration of AI could help build stronger and more meaningful clinical relationships. This perspective is consistent with recent findings that highlight the potential of AI to support more compassionate care by relieving professionals from routine tasks [43].

The perspective on the potential applications of AI contrasts with reality, as few tools are currently being implemented in primary care [44]. Stakeholders expressed concern that prematurely adopting these tools could impose a significant workload on health care professionals. Successful integration will require time to adapt to these technologies and ensure that they are safe and effective for both patients and health care professionals.

### Data as the Cornerstone of AI Development

A previous qualitative meta-synthesis that reviewed public perceptions concluded that the public has reservations regarding the use of medical AI, including those pertaining to security, privacy protection, and legal responsibility [12]. Similarly, in our study, primary care physicians expressed growing concern over the potential adverse consequences of sharing patients’ private information to develop data-driven tools. These concerns include the risks of unauthorized use of personal information by companies driven by purely economic interests. Such risks have led some patients to resist sharing their data, demanding complete anonymization and continuous transparency regarding how their personal information is used and by whom.

The literature on information privacy presents a variety of methodologies for the study of privacy violations that have been categorized as reidentification, reconstruction, and property inference attacks. An alternative approach involves promoting privacy-preserving machine learning techniques, which are intended to enhance the security of these applications and prevent the occurrence of such attacks [45].

Several political entities are developing regulations to address the challenges of managing patient data for AI-driven technologies. The European Union has taken a leading role with the enactment of the AI Act [46] and the adoption of the European Declaration on Digital Rights [47], establishing a comprehensive framework for ethical AI development that emphasizes transparency, accountability, and the protection of fundamental rights. In contrast, the United States has proposed an AI Bill of Rights that focuses on safety, nondiscrimination, privacy, transparency, and human oversight. However, this framework remains a nonbinding proposal, and its implementation remains uncertain [48].

### Barriers and Facilitators Regarding AI in Primary Care

The review suggests that the acceptance of AI in health care largely depends on specific patient characteristics, including age, prior experience with technology, technological affinity, and SES. In particular, SES has been identified as a significant barrier to accessing the benefits of AI, with the risk of further widening the gap for communities historically marginalized from health care services and, eventually, increasing health inequities [49]. Furthermore, racial and ethnic disparities in health care quality are well documented. Current AI tools are predominantly trained on data from specific populations, such as White people, men, or nonpregnant women. This fact may introduce biases when applied to diverse demographic groups, potentially compromising the accuracy and fairness of AI-driven health care interventions [50]. These findings are consistent with other studies that emphasize the importance of considering race, ethnicity, and gender in achieving equitable health care outcomes [51,52].

Health care providers have identified several essential elements that future tools must incorporate to ensure effective implementation. Key considerations include affordability, intuitive design, and adaptability to physicians’ needs. Furthermore, health care providers stressed the need for early and ongoing involvement of all relevant stakeholders, beginning at the conceptualization stage and persisting throughout the project life cycle. Without such sustained engagement, these tools may fall short of addressing practical challenges and user requirements in real-world health care settings.

### Implications and Significance of the Findings

To the best of our knowledge, this constitutes the first systematic review in which findings from qualitative studies examining perceptions of AI in primary care settings were synthesized. The qualitative component provides insight into the perceptions and attitudes of all stakeholders within primary care settings, which facilitates the formulation of practical and evidence-based conclusions.

Studies focused on primary care enable a more detailed understanding of the distinctive characteristics inherent to this domain of medicine. Consequently, this review can guide the ethical, safe, and effective implementation of AI in primary care settings.

### Limitations

This meta-synthesis has some limitations that should be acknowledged. First, only studies published in English and Spanish were included, which may cause language bias. Second, the review encompasses studies that extend beyond an investigation of the perception of a particular profession; instead, it includes research involving a range of stakeholders, including physicians and primary care patients, but further research is required on other primary care professionals, including nurses and assistants. Third, heterogeneity is present in the data collection and the interpretation methods used, as well as in the sample recruitment strategies used. Fourth, although it would have been highly desirable to include a study conducted in Spanish, only 1 study by Catalina et al [53] addressed this issue; however, it was a survey based on closed-ended questions and therefore did not meet the inclusion criteria. Fifth, differences in the conception of the primary care physician’s function exist, with competencies varying depending on the country where the profession is practiced; however, these variations may contribute to differing perspectives on the issues being studied.

The final search strategy did not include terms related to clinical decision support systems. Similarly, other AI tools not described or classified as machine learning or AI were not included, as adding these keywords to the search would have significantly increased the number of results, making them difficult to interpret. Finally, some studies lacked a sufficient explanation of the theoretical framework and did not clarify how the researchers’ backgrounds were addressed.

### Conclusions

To the best of our knowledge, this is the first meta-synthesis to suggest that, from a primary care perspective, medical AI is a tool with the potential to improve health care significantly but also to introduce several social and ethical challenges into the discourse. The introduction of AI in primary care settings has the potential to assist physicians in their work, but it has also raised concerns about the role of physicians in decision-making processes and the security of patient information entrusted to both the physician and the system.

In this paper, we present recommendations for the integration of medical AI in primary care settings, based on the insights of various stakeholders in the field. It is imperative that AI is developed in a way that respects the full range of contexts that converge within primary care settings. Ensuring these conditions guarantees that the technology is as fair and accurate as possible.

## Appendix

Multimedia Appendix 1 ENTREQ checklist.

Multimedia Appendix 2 Search strategy for each database.

Multimedia Appendix 3 Joanna Briggs Institute Critical Appraisal Checklist for Qualitative Research.

Multimedia Appendix 4 Quotations by themes.

Multimedia Appendix 5 Evidence profile with the Grading of Recommendations Assessment, Development, and Evaluation (GRADE)-Confidence in the Evidence from Reviews of Qualitative research (CERQual) assessments.

Multimedia Appendix 6 PRISMA (Preferred Reporting Items for Systematic Reviews and Meta-Analyses) checklist.

## Abbreviations

AI: artificial intelligence
eMERGe: Evidence-Based Measures of Empowerment for Research on Gender Equality
ENTREQ: Enhanced Transparency in Reporting the Synthesis of Qualitative Research
GRADE-CERQual: Grading of Recommendations Assessment, Development, and Evaluation–Confidence in the Evidence From Reviews of Qualitative Research
SES: socioeconomic status
SPIDER: Sample, Phenomenon of Interest, Design, Evaluation, and Research Type
