# Supplementary material for: Perceptions of, Barriers to, and Facilitators of the Use of AI in Primary Care: Systematic Review of Qualitative Studies
Source: J Med Internet Res. 2025 Jun 25;27:e71186. doi: 10.2196/71186 (PMC12242059; doi:10.2196/71186)
Supplement: Multimedia Appendix 2 [file jmir_v27i1e71186_app2.docx]

## Search strategy for each database

*Date: 09^th^, June 2024*

### PUBMED

| **S1** | ((attitude[MeSH Terms]) OR ("attitude*"[Title/Abstract]) OR (concern*[Title/Abstract]) OR (perception*[Title/Abstract]) OR (perspective*[Title/Abstract]) OR (qualitative research[MeSH Terms]) OR (“qualitative research” [Title/Abstract]) OR (interview*[Title/Abstract]) OR (“qualitative inquiry” ) OR (“qualitative stud*”) OR (“focal group*”)) |
| --- | --- |
| **S2** | ((primary health care[MeSH Terms]) OR (“primary health care”[Title/Abstract]) OR ("primary care*"[Title/Abstract]) OR ("family medicine"[Title/Abstract]) OR ("family practice"[Title/Abstract]) OR (rural health[MeSH Terms]) OR ("rural health*"[Title/Abstract]) OR (general practice[MeSH Terms]) OR (“general practice*”[Title/Abstract]) OR (general practitioner[MeSH Terms]) OR ("general practitioner*"[Title/Abstract]) OR (primary care physician[MeSH Terms]) OR (“primary care physician”[Title/Abstract]) OR (nursing, primary care[MeSH Terms])) |
| **S3** | ((ai artificial intelligence[MeSH Terms]) OR ("artificial intelligence"[Title/Abstract]) OR (neural network[MeSH Terms]) OR (“neural network” [Title/Abstract]) OR ("computational intelligence"[Title/Abstract]) OR ("medical big data") OR ("healthcare data"[Title/Abstract]) OR ("data sharing"[Title/Abstract]) OR ("deep learning"[Title/Abstract])) |
| **S4** | S1 AND S2 AND S3 |

*Results:* 456

### Web Of Science

| **S1** | (“Artificial intelligence” OR “AI” OR “medical big data” OR “healthcare data” OR “data sharing” OR "computational intelligence" OR "deep learning" OR “neural network”) |
| --- | --- |
| **S2** | (“Primary Care*” OR “family medicine” OR “family practice” OR “Rural Health*” OR “general practitioner*” OR “general practice” OR “primary care physician”) |
| **S3** | (“qualitative inquiry” OR "qualitative research" OR “qualitative stud*” OR “focal group*” OR “interview*” OR concern* OR “attitude*” OR “perception*” OR perspective*) |
| **S4** | S1 AND S2 AND S3 |

*Results:* 667

### SCOPUS

| **S1** | TITLE-ABS-KEY (“Artificial intelligence” OR “AI” OR “medical big data” OR “healthcare data” OR “data sharing” OR "computational intelligence" OR "deep learning" OR “neural network”) |
| --- | --- |
| **S2** | TITLE-ABS-KEY (“Primary Care*” OR “family medicine” OR “family practice” OR “Rural Health*” OR “general practitioner*” OR “primary care physician”) |
| **S3** | TITLE-ABS-KEY (“qualitative inquiry” OR "qualitative research" OR “qualitative stud*” OR “focal group*” OR “interview*” OR concern* OR “attitude*” OR “perception*” OR perspective*) |
| **S4** | S1 AND S2 AND S3 |

*Results:* 473

*TOTAL results:* 1596

*Results after duplicated removed:* 942 (with Rayyan intelligent systematic review app).
