# Supplementary material for: Perceptions of, Barriers to, and Facilitators of the Use of AI in Primary Care: Systematic Review of Qualitative Studies
Source: J Med Internet Res. 2025 Jun 25;27:e71186. doi: 10.2196/71186 (PMC12242059; doi:10.2196/71186)
Supplement: Multimedia Appendix 3 [file jmir_v27i1e71186_app3.docx]

## Joanna Briggs Institute (JBI) Critical Appraisal Checklist for Qualitative Research

| **Reference** | **Q1** | **Q2** | **Q3** | **Q4** | **Q5** | **Q6** | **Q7** | **Q8** | **Q9** | **Q10** |
| --- | --- | --- | --- | --- | --- | --- | --- | --- | --- | --- |
| **Darcel et al [27] 2023** | Y | Y | Y | Y | Y | Y | Y | Y | Y | Y |
| **Upshaw et al [28] 2022** | Y | Y | Y | Y | Y | Y | Y | Y | Y | Y |
| **Terry et al [29] 2022** | Y | Y | Y | Y | Y | Y | Y | Y | Y | Y |
| **Nash et al [30] 2023** | Y | Y | Y | Y | Y | Y | Y | Y | Y | Y |
| **Allen et al [31] 2024** | Y | Y | Y | Y | Y | U | Y | Y | Y | Y |
| **Richardson et al [32] 2022** | Y | Y | Y | Y | Y | U | U | Y | U | Y |
| **Richardson et al [33] 2021** | Y | Y | Y | Y | Y | U | U | Y | U | Y |
| **Kocaballi et al [34] 2020** | Y | Y | Y | Y | Y | Y | U | Y | Y | Y |
| **Fraile-Navarro et al [35] 2023** | Y | Y | Y | Y | Y | U | Y | Y | Y | Y |
| **Buck et al [36] 2022** | Y | Y | Y | Y | Y | U | Y | Y | Y | Y |
| **Kamradt et al [37] 2022** | Y | Y | Y | Y | Y | U | Y | Y | Y | Y |
| **Blease et al [38] 2019** | Y | Y | U | Y | Y | U | Y | Y | Y | Y |
| **Mikkelsen et al [39] 2023** | Y | Y | Y | Y | Y | Y | U | Y | N | Y |

Y: YES; N: NO; U: UNCLEAR.

Q1. Is there congruity between the stated philosophical perspective and the research methodology?

Q2. Is there congruity between the research methodology and the research question or objectives?

Q3. Is there congruity between the research methodology and the methods used to collect data?

Q4. Is there congruity between the research methodology and the representation and analysis of data?

Q5. Is there congruity between the research methodology and the interpretation of results?

Q6. Is there a statement locating the researcher culturally or theoretically?

Q7. Is the influence of the researcher on the research, and vice- versa, addressed?

Q8. Are participants, and their voices, adequately represented?

Q9. Is the research ethical according to current criteria or, for recent studies, and is there evidence of ethical approval by an appropriate body?

Q10. Do the conclusions drawn in the research report flow from the analysis, or interpretation, of the data?

**References**

20. Darcel K, Upshaw T, Craig-Neil A, et al. Implementing artificial intelligence in Canadian primary care: Barriers and strategies identified through a national deliberative dialogue. Mohammadzadeh A, ed. PLoS One 2023;18(2):e0281733. doi:10.1371/journal.pone.0281733

21. Upshaw TL, Craig-Neil A, Macklin J, et al. Priorities for artificial intelligence applications in primary care: a Canadian deliberative dialogue with patients, providers, and health system leaders. J Am Board Fam Med 2023;36(2):210-220. doi:10.3122/jabfm.2022.220171R1

22. Terry AL, Kueper JK, Beleno R, et al. Is primary health care ready for artificial intelligence? What do primary health care stakeholders say? BMC Med Inform Decis Mak 2022;22(1):237. doi:10.1186/s12911-022-01984-6

23. Nash DM, Thorpe C, Brown JB, et al. Perceptions of artificial intelligence use in primary care: a qualitative study with providers and staff of Ontario community health centres. J Am Board Fam Med 2023;36(2):221-228. doi:10.3122/jabfm.2022.220177R2

24. Allen MR, Webb S, Mandvi A, Frieden M, Tai-Seale M, Kallenberg G. Navigating the doctor-patient-AI relationship - a mixed-methods study of physician attitudes toward artificial intelligence in primary care. BMC Prim Care 2024;25(1):42. doi:10.1186/s12875-024-02282-y

25. Richardson JP, Curtis S, Smith C, et al. A framework for examining patient attitudes regarding applications of artificial intelligence in healthcare. Digit Health 2022;8:205520762210890. doi:10.1177/20552076221089084

26. Richardson JP, Smith C, Curtis S, et al. Patient apprehensions about the use of artificial intelligence in healthcare. NPJ Digit Med 2021;4(1):140. doi:10.1038/s41746-021-00509-1

27. Kocaballi AB, Ijaz K, Laranjo L, et al. Envisioning an artificial intelligence documentation assistant for future primary care consultations: A co-design study with general practitioners.  J Am Med Inform Assoc 2020;27(11):1695-1704. doi:10.1093/jamia/ocaa131

28. Fraile Navarro D, Kocaballi AB, Dras M, Berkovsky S. Collaboration, not confrontation: understanding general practitioners’ attitudes towards natural language and text automation in clinical practice. ACM Trans Comput-Hum Interact 2023;30(2):1-34. doi:10.1145/3569893

29. Buck C, Doctor E, Hennrich J, Jöhnk J, Eymann T. General practitioners' attitudes toward artificial intelligence-enabled systems: Interview study. J Med Internet Res 2022;24(1):e28916. doi:10.2196/28916

30. Kamradt M, Poß-Doering R, Szecsenyi J. Exploring physician perspectives on using real-world care data for the development of artificial intelligence–based technologies in health care: qualitative study. JMIR Form Res 2022;6(5):e35367. doi:10.2196/35367

31. Blease C, Kaptchuk TJ, Bernstein MH, Mandl KD, Halamka JD, DesRoches CM. Artificial intelligence and the future of primary care: Exploratory qualitative study of UK general practitioners’ views. J Med Internet Res 2019;21(3):e12802. doi:10.2196/12802

32. Mikkelsen JG, Sørensen NL, Merrild CH, Jensen MB, Thomsen JL. Patient perspectives on data sharing regarding implementing and using artificial intelligence in general practice – a qualitative study. BMC Health Serv Res 2023;23(1):335. doi:10.1186/s12913-023-09324-8
