# Supplementary material for: Perceptions of, Barriers to, and Facilitators of the Use of AI in Primary Care: Systematic Review of Qualitative Studies
Source: J Med Internet Res. 2025 Jun 25;27:e71186. doi: 10.2196/71186 (PMC12242059; doi:10.2196/71186)
Supplement: Multimedia Appendix 4 [file jmir_v27i1e71186_app4.docx]

## Quotations by themes

*Theme 1: change in the physician-patient relationship*

##### AI does not understand feelings [21–24,27–29,31,32]

*Technology cannot replace doctors. There is definitely a 6th sense.*

*It is unlikely that the human element of empathy and the subtlety of human communication and non-verbal cues can be detected by robots or machines.*

*So much of ill health is vague, complicated and psychological, and the lack of IT to any time soon pick up on individualized, non-verbal cues etc. I feel will still leave a huge role for people.*

*I know that people talk about AI replacing clinicians. I don’t think that’s where we are at the moment. I still think that clinical acumen, that knowledge, that contextualizing it for the patient is quite important. But the technologies themselves, [. . . ] it would actually make you quite efficient, make the working day more enjoyable that you have more time to spend with the patient and to contribute to patient care.*

*Technology will never attain a personal relationship with patients. We are essentially a people business. It’s personal relationships that count.*

*They [patients] are humans and subject to the vagaries of human recall, memory, and interpretation. AI may make it easier to interpret a blood result follow a protocol or order a test. But AI will always struggle when the same human can score 1/10 for a symptom today and 10/10 tomorrow.*

*[AI-enabled systems] are not stand-alone systems but are networked, and [...] actually, work over the internet with such simple things as voice recognition. And in my view, this will change the doctor-patient contact considerably. [...] I consider the fundamental trust in the patient-physician-conversation [...] to be a very important basis for our work. And I also see [the trusting relationship between the patient and the physician] as being in danger due to the increasing use of such procedures. I find this very worrying.*

*Medicine, particularly general practice, is an art; listening to ideas concerns and expectations and negotiating a shared plan with the patient. Often doing nothing other than listening is required. I wonder how well a computer will be able to do this?*

*Technology won’t replace GPs as patient management is about negotiation and managing risks and different patients have different views.*

*There is something behind almost every illness that makes [diagnosis] even more challenging. And if this is not considered, it will not be possible to help a patient comprehensively. And I think [AI] can probably not do this.*

*Experience can hardly be replaced by AI. Experience and intuition. And empathy. This is just how I treat people, to get something out of them. So, this is something that defines a good physician and cannot be replaced by AI. Empathy.*

*Yeah, AI will never be able to do that… reading the body language, reading emotions, the longstanding relationship you have with that patient. So that has to be seen as always being a peg above what AI could do.*

*Electronic health records have advantages for sure…But…the one thing I miss so much [is] that I can no longer do a genogram. There's nothing. They were never designed [for EMRs]. So, my family histories are so different than how I was taught with that picture. I did it with patients and we could really understand their family history from so many different angles. I used to love that part of care…We just have to be careful [with AI so] that we don't lose…strengths of [the] older model.*

*How does AI…consider the triad of, you know, what is the evidence? What is my experience that I've had after 35 years in practice? And what are the patient preferences?…I'm not sure how AI could pick up understanding my clinical experience. I don't know how AI can pick up what a patient's preferences [are] either. So, I think [of AI] as a tool…within our evidence-based medicine model.*

*Because increasingly the technology means you can do a lot of things without the patient actually being in front of you. That is confronting to the traditional model … The [healthcare] system doesn't allow that … you've got to be face-to-face with the patient.*

*I think the point of the doctor is to give suffering meaning. It's to provide a steady hand, I think. It's to support people through issues … a computer can support you, but it doesn't have any meaning because there's no emotional risk from the computer because the computer does not know what it means to live or die.*

*People are certainly beaten by [AI] in many ways. But not in the emotional one.*

*The patient comes in but then you're asking how their husband is who's never been in to see you but doesn't want to, but you then try to help their health care as part of that consultation. I mean this [the AI assistant in the video] has no way of … it's that caring bit … because you see the body language, so sometimes it's not what they say, it's something you pick up … the computer won't be able to pick that up.*

*I think undifferentiated illness would be tricky with this … You know if the patient that comes in—“oh, I'm just feeling a bit tired or my appetite is not great”—very undifferentiated things … I mean something that's very … protocol driven … It would be very easy to do with this system [the AI assistant in the video], but I would imagine it would be much harder with a patient that comes in with kind of vague things which often we see as GPs.*

*Sometimes … patients will say … a jumble of things. Part of our job's to pick up the significant things, but in this sort of system [the AI assistant in the video] everything is picked up, and something might seem irrelevant at the time, but then six months later it's like, oh, that was actually a huge thing.*

*I’ve known a lot of my patients now for 30 years and know a lot about them. That can’t really be put into a data set that AI can draw upon.*

*I think there are a lot of people who think that they know more than the computer or that there's that human element that the computer doesn't have. That gut feeling that the doctor has that, this is what it is. Well, you can't get that with AI.*

*Also, if we rely more and more on technology, it will also cause clients to say, “I don't even talk to my provider anymore.”…It takes time for our clients to share information. And at times you'll see they're withholding a lot of information that's quite important. And then once there is trust they'll say a lot of things. And so, if we want to provide good care from the very beginning, losing [the trusted relationship] is going to delay care that the clients would need.*

*Maybe you are actually then more compassionate in an encounter, because you haven’t had to do all of that mental lifting.*

*In that situation one will feel pressured to please the GP and say, “oh yes of course”.*

*I guess over time as it improves, it’s more precise, fewer errors, and there’s more trust in the system, then yes, a lot more. . . Maybe there could be a bit less checks and balances.*

*If there are technologies developed, and you’re first working with them, you probably do want to have that control.*

*I suppose it is that balance isn’t it, I still want to have control over some things, and it’s because I know that the sort of information that comes into our system, there just isn’t enough information there for it to be able to happen automatically.*

*I'm much more comfortable with AI as an assistive tool, rather than making decisions on its own.*

*You're destroying social contract with this [AI assistant]*

*Why not just record the whole thing and just be done with it, and never alter it.*

*Because the machine's doing all the flying so the pilot's there just to make sure everything is okay. … Ultimately, the human is there to make sure that if there is a discrepancy that your experience comes in.*

##### Patient preferences [20,21,25–27,29,31,32]

*Technology will never be satisfactory as patients are looking for that interaction and dopamine squirt (doctor is the drug) which can only be achieved through empathic continuity of care of highly experienced General Practitioner specialists.*

*My experience every day with patients is that they want to be touched, and they want to look you in the eyes.*

*I want my doctor to be present with me in a conversation and not staring at the computer and looking at all the tools or boxes that they need to check off.*

*When I'm struggling [to manage my diabetes], sometimes…I'm just tired of being a diabetic. It's not because…I don't know how to take care of myself…It’s only when a trust relationship has been built up with the doctor that he can begin to say, “Okay, I know you know how to take care of it. You don't seem to be taking care of it right now. What's going on for you?” I think you have to rely…on the trust relationship between the doctor and the patient to recognize specifically what's going on. It may be more subtle than the things that AI might pick up.*

*In my case, I can tell you for sure that an AI would say, “Oh, she needs this prescription.” Meanwhile, that could kill me…There are nuances here that I don't think an AI could know…And look at it this way: in finance, we have controls in place…So, I would want the doctor to review that first. I'm all for them not having to do as much typing. I think it would save time, but there would have to be that review.*

*I would like to trust that my provider has a good perspective in AI so that he doesn't just sort of follow it slavishly but considers it as part of his care for me.*

*You know what, that’s just like for me, I have multiple sclerosis, but I also have fibromyalgia, so the thing is when I hurt, I can’t tell you why it hurts, you know what I’m saying? I can’t tell you, oh my fibromyalgia’s kicking up or I’m having a MS attack or whatever. I can’t tell you the difference, and if I can’t tell you the difference, I know AI cannot tell me the difference.*

*Well I think of this August… all of a sudden, I’m getting headaches, and they weren’t going away, and I don’t like pills, so I was avoiding, and all of a sudden they were getting to the point, so I got on the phone with a nurse and she asked me a thousand questions, and she says, you wanna come in today or tomorrow? Ya gotta know if this question’s answered to get to the next… where you go with the next question. I don’t know if the robot would… artificial intelligence know, okay, this one answered yes, so we gotta go this direction or.*

*You have trust towards your GP, and they are kind of an authority person, they know best, and you have to trust that. It is also a form of power.*

*A person does not have a trustful relationship with a machine.*

*I’d be okay with them telling a doctor what to do, but I don’t know that I’d want a machine doing the treatment, especially depending on what it is. Aiding, sure, they already do that with robotics and CT scans and all that, but I want a human there making sure that it’s doing what it’s supposed to.*

*So it’s your . . . how much do you trust this AI tool, but I can also say how much do I trust the existing healthcare system? And in both of those, it’s . . . when you’re really ill, you have to trust it ‘cause there’s no choice.*

*That’s not what she wanted to hear, so she went to another doctor, and she went to … several doctors because she knew there was a problem and she was going to find a doctor who confirmed what she knew. Now if there had been, or she had accepted some artificial intelligence, maybe that would’ve helped her.*

*I feel good about it. I think it has the ability to be better. I mean, it’s not a human. It’s got more data, so probably. … [I]t probably has more intelligence; it just has more information to work with to try to come up with a proper diagnosis. … I don’t think you will cure a lot of diseases without that advanced intellect. Obviously, we’ve come a long way with the human brain, but we could probably go a lot farther and speed the process with AI.*

*They [patients] can do it [have the consultation] at home. You feel like rubbish in the morning, do you really want to have to call the GP, get an appointment, sit in a waiting room for an hour with other coughs and colds … think of all that disease that's spread in a waiting room.*

*Yeah, that's probably better than what they're already doing.*

*Some people are not great at doing mental health consults … there are some people that are terrible and for those people just the right words …*

*[With] AI, you can then understand how [the physician] came to a decision because AI said the risk was 0.001.*

##### Physicians´ concerns [20,22–24,27,29]

*… there is that downside as well if we offload everything to the machine if it were possible, we would also become worse in another way. We would lose our intellectual capacity…*

*I think eventually it [AI] will take all the jobs. We're going to be the last group anyway to use this.*

*I think we can be influenced [by the patients’ opinions] because, in the end, a medical practice follows the market like a small business. If the patients want [AI technologies] and demand [AI technologies], more and more practices will offer it.*

*At one point, the own decision and the own expertise threatens to be pushed into the background or to become redundant.*

*Surely, many doctors probably see their unique medical status endangered, that they are under the surveillance of others, that they think there is a bit of an attack on their own vanity.*

*If they [patients] think that we're just getting suggestions from a computer, then maybe they can just get suggestions from a computer. I think it becomes more difficult to convince them that our recommendations are more valuable than what they can pick up on the internet.*

*Patients may end up feeling that, you know, if the AI can tell me that then why did I bother to come to you?*

*The more we sort of train patients to expect things quickly and efficiently, the more expectations are on the doctor to then produce in the same way.*

*My greatest fear is that you lose your critical thinking because something’s going to come up on a screen and tell you what to do.*

*we don’t want to lose that ‘art of medicine’ that we have learned over the years, because it’s a very challenging profession where you see people with undifferentiated complaints, so you have to know something about everything. And with AI, if we rely too much on it, we might lose that clinical judgment.*

*Since [the patient] has the feeling [...] that the machine takes care of it and the doctor would only have to put his signature under it.*

*[The treatment] may drift off into a standardized interview, and that’s probably not necessary.*

*I think eventually the doctors will be the assistant doctors … Doctors will assist artificial intelligence what to do … eventually … we'll be helping it. I think we'll be assistant … Because they'll be doing everything. It will be just saying, yes, no, yes, no. Say supervision, but we'll be assisting.*

*I’m worried about AI, introducing a dynamic where misinformation is enhanced… if a patient comes in and they’re like, hey, like, you know, WebMD.GPT told me that I need an MRI then there’s another powerful thing that I’m arguing against.*

### *Theme 2: AI as a partner for efficient time and information management*

##### The physician’s partner [20–23,25,27–29,31,32]

*Machines should be good at initial triage of uncomplicated patients presenting to primary care.*

*My sense is that [triage in primary care] is absolutely abysmal…Like, there is no function, currently, of sophisticated pre-visit triage…In [my] clinic, people wait 20 minutes and hang up. They don't even call because it's so hard to get in. That's our triage system. If [there was] a way to…symptom check or…prescreen a little bit, they might be more likely to [come in].*

*[AI must be] scientifically grounded and must provide validated results that [the physician] may not be able to produce in their entirety.*

*Now a completely new virus has appeared in China or Japan, and to get ahead of it, you need artificial intelligence which can detect [the virus] much faster.*

*If AI is well programmed or if there are no failures in it, then AI is more accurate than a person, who is sometimes tired [and thus] makes bad decisions.*

*I feel like when AI is making those kinds of contributions where it’s actually identifying things that humans would not identify. And that’s in front of people; they can see it, they can say ‘You know what, I just missed a breast cancer diagnosis and this tool caught it.’ That’s when things start to become really interesting.*

*Maybe AI could help keeping track of patient records… AI could get an overview fast and see that this patient has now shown these symptoms for the fifth time, so maybe it is time to look into that instead of the GP missing it.*

*If a general practitioner has patient data from a person for many, many years and predictive analytics can analyze that data and say ‘OK, over the past 10 years this person’s blood pressure has been steadily increasing, maybe we should act on this now’ I think that will be a really useful tool for healthcare providers to have.*

*They might come back with okay, these are the tests that need to be run. Then you go and get those tests run, and they input that in, and then they can pinpoint and be more accurate with their diagnosis then, and I could see how they would be more accurate, really, with knowing everything’s that ever been known about modern medicine and all the cases out there and collating all the data, so I could see how they would be more accurate.*

*I had a strange illness 10, 11 years ago, and I went through PET scans and CT scans and the whole 9 yards over and over and over and over again for 6 months, and I was miserable with it, and what they ended up doing, they had to take out part of my lung to find out what it was, and if there was some type of AI out there that could have helped in that… [I might have] skipped 6 months’ worth of trial and error.*

*But for rarer diseases, when it comes to making a diagnosis; for example, a red skin spot that I can’t classify at all, then it would be conceivable [...] to reaffirm or reassure oneself [by means of AI].*

*If you requested all the notes from their previous GP, for example, and then I guess you wanted the system to analyse, and create a brief summary, or transcribe that information into the electronic medical record. I think the greatest use would be to. . . Like for new patients, their notes from their previous GP to naturally transcribe that into your electronic medical record.*

*I think it’s good to have the more input. The more input that comes into here, the better I think it’s going to be able to respond to a situation. We definitely all agree, I think, in this room that the more information that is collected, the better off all people are gonna be . . .*

*I think it’d be very helpful if it could provide a clinical handover summary of the patient almost with the key person and features that you’re normally looking for.*

*For example, the doctor in the previous consult records this significant problem and then you say, “Okay, can you bring up some significant past medical history?” Then you should be able to filter out because most notes are quite huge, it will filter out and bring up the important things. And that probably will be useful before you start to see the patient. Maybe there should be some way for a doctor to mark this as important so that it brings it up and the AI can bring it up later on when another doctor looks at the notes.*

*It may be useful to say “can you bring up the discharge summary from so and so date or for so and so admission”. I think that will be useful.*

*One of the things that people want to look at also is to be able to summarise a consultation, which is held in natural language, into a summary letter without anyone needing to type anything.*

*I thought might be actually really helpful if, at the end of a consultation it[the system] automatically sends the patient a summary of the plan. I guess one of the things that you often tell patients is you talk to them about. . . If they’ve got a cold and if you don’t think it’s COVID, we might say, try some warm steam, some days we’ll say, ... if that just gets automatically emailed to the patient, that will be amazing.*

*And hopefully detecting the keywords, like particular symptoms, and signs, and duration, and quality, and characteristics of what the patient’s telling. And then translating that into a succinct, concise medical note that is similar to what a doctor would write for their notes.*

*If there were quick ways of extracting some key things, like key diagnoses, the medications, who their specialists are, and that’s then in your notes, . . . That would be helpful.*

*Trying to remember all the guidelines and having to look in so many spots, I think that creates a lot of mental fatigue [for the provider].*

*GPs receive discharge summaries or letters from specialists as like PDF documents. So it’s not really possible to search those documents for key terms or keywords. I’m wondering whether there may be some opportunity to be able to search documents. Search for things within documents and so then that can help if a patient comes in with an undifferentiated presentation, to be able to better understand what has previously occurred.*

*One of the advantages of when you write it is it reinforces what you thought … It's a thinking process, because you actually think about what this actually means? … How can you capture that writing experience in an electronic medium?*

*Once you trust the system, if it could just automatically do all of that [extracting information from discharge summaries], would be great. So, add new medications, add classifications or diagnoses.*

*And also, something that I didn’t mention earlier is also if there’s any way to quickly search or locate information. Because there are times like, I don’t even know, a patient comes in and is it must be in the notes.*

*Imagine if a patient's sitting in a practice … if they get given an iPad that has … a written assistant that says—“Can you tell me a little bit about why you're here?”—The patient types in their presenting complaint … any family history of conditions, any current medications … When that patient comes in to see me, that information is presented to me in a manner that I can copy and paste, and I can go through it.*

*If there was a Tom's visit summary, so I could print off a document saying, Tom, you've come in with cough … Here's what I think's going on. Here's the management plan and here [are] the things to look out for and come back and see me … if one of these happen.*

*You just tell the computer what [you] want to check and then the computer will do the job and generate the report.*

*The most useful application of that would be for the natural language processing to read the discharge summary. And then suggest what could be updated in the patient’s file. If the natural language processing may pick up a dose change, or a change in medication, then it would suggest updating in the EMR or electronic medical record. And the doctor would just approve it, or edit it by. . . To make it faster that way.*

*It would be very useful to compare the text to what’s in the file and make adjustments or have options to make easy adjustments. Entering medications is actually quite a cumbersome job, and so anything that could help with that would also be a valuable thing.*

*I guess it’s an idea that I’ve had to be able to automate the automatic cataloguing of correspondence that comes through in general practice. For example, exactly as you’re saying, discharge summaries, specialist letters, lab results, if it comes in, it’s automatically categorized because there are some letters that are pretty much useless.*

*There'd be an audit trail around the system. You remove something you didn't think was important … but it may have actually been important. This way it's your judgement and your skills as to what you put in the record.*

*The summary of the consultation, it could certainly help, it could certainly be useful there.*

*I quite like the idea of having an app that's safe, that patients can update their health information that can link to the medical records.*

*The machine start[s] to learn your behaviours and is more predictive to your style … [the machine] that you're using is more nuanced to your individual style.*

*Be useful to develop AI to do analyses of pathology returns, and read all the letters, to provide another presence in the consulting room, and to write the referral letters, organize investigations and the like, ie, act like a personal assistant might do.*

*I think technology’s place is more about informing patients about conditions and management booking appointments ordering prescriptions contacting the surgery via the internet rather than the phone.*

*Wouldn't it be amazing if the AI could capture all that advanced care directives and all [the] irrelevant stuff. You want to take away mundane repetitive tasks.*

*I think they have the potential to be incredibly useful. Particularly I guess where I’m thinking you’re coming from is in relation to medical records. So one of the problems I find as a GP and what a lot of colleagues tell me as GPs. Is that they struggle to see patients and then record their medical notes after the consultation, within the 15 minutes that they have. So any technology that allows GPs to more accurately record records in a shorter period of time would be incredibly useful.*

*The more I read about AI and the more I see the selling point is that physician burnout piece. And I know it’s across every sector but in CHCs too because they’re underpaid, they’re not getting the increases, they are paid salaries—there’s nothing they can do to work harder or to get more money. But kind of framing it in that “Oh this is going to help you. We understand you’re suffering.*

*I think the goal of medicine is to either heal you or to live a more fuller, richer life, and I think if AI with all its knowledge can help do that, if it can help me get better, it’s like that extra little thing that . . . you know, that extra training that’ll make me a better runner, that extra math class that’ll help make me smarter in math. It’s just that little extra that maybe will achieve that goal of, like I said, leading healthier lives.*

*We are only humans, so is the GP. Making an extra check with AI would make me feel safer.*

*I suppose in some countries where you're managing maybe thousands and thousands of people and having oversight, where the individual things are being managed by these [AI] assistants … those things can really give high quality medicine to low resourced countries. Now, they'll make mistakes, but even, you can bear those mistakes, basically, because of the overall benefit of them.*

*You know if we think about how many prescriptions we write every day… how much data we’re feeding into a system, test results that are coming... I’ve got to manually process that stuff right now and its super time-consuming, laborious and unfortunately there’s no better way to do it, so I’ve got to do it...it feels like it takes up an hour or two of every day and if I had something, a machine, that could do that for me in an accurate way and alert me when there’s something that I need to look at, like it’s red-flagging things in real time then that would be a huge breakthrough because then I could identify patient safety issues as they happen.*

*It’s maybe like a second opinion right there. Maybe imagine you have the professor of general practice sitting there with you, guiding you in your consult. What an amazing experience that would be for both the doctor and the patient.*

*Can you imagine [if] the computer gives a suggested empathetic statement and you read it out—“oh, that must be really difficult what you're going through.*

*It will make life easier, and it will integrate and maybe improve care for the patient, that is what I’m thinking.*

*I mean what we know now and we think of what we thought we knew 50 or 100 years ago in medicine, and it’s just gonna keep building and building, and the potential to have that added resource to do that is a huge opportunity. It wasn’t that long ago that they used leeches, so. . . you know what I’m saying? I mean really in the span of time and how much exponentially we’ve learned about conditions . . .*

*The nice thing about this [the AI assistant in the video] is as an individual practitioner, you'll be able to do it, so it will be our option … It's going to be a bottom up kind of diffusion not a top down dinosaur, so the barriers to doing it [are] going to be much easier … because we'll find the clinical utility of it and want to adopt.*

*…they [patients] want to know why ...their hemoglobin is off by one point… actually, AI might be a solution for that, right? If it can give them answers to things they want to know. Again, back to that self-care, chronic disease management, I think as long as it’s as authoritative or as peer reviewed as the current literature allows or suggests.*

##### Time matters [20,21,24,25,27–30]

*As a patient, I don't want my doctor spending his time facing the computer. I want him facing me…So in terms of looking at all of those admin tasks that are taking away from the patient care, I think AI has the potential to free up that time so that I have more face time with my doctor.*

*I was struck by the overlap in interests between providers and patients…What stood out was creating more time to be able to focus on actual patient and provider interaction… just having more of that time not taken up by all these other nonclinical issues. That…could represent a very safe, low-risk place to start and to sort of build upon AI within the primary care setting.*

*Exactly, more time for patients, then better decisions to improve mortality and morbidity of patients. Exactly, I believe these are the two most important aspects.*

*Yes, just taking my hands off the computer, getting my eyes off the screen, so that I can be spending time with the patient. And also saving me the documentation time, because you can either spend more time with the patient or see more patients.*

*It wouldn’t be like imagine, like, in five years or ten years’ time, it will be like, before the patient enters, like “show me a summary” or “show me significant medical issues” or “what did we deal with in the last medical consultation?” or “what changes did we do” Something like that. And then once the patient comes in, maybe it should take notes automatically without me needing to type it.*

*Please hurry up with the technological advances to take away some of the crap that I still have to sort out–then I will be able to get back to proper diagnosing and doctoring.*

*I think that doctors are often overwhelmed and overworked and if AI can be used to help with that, I'm all for that, so that they can be more efficient and more effective in their work.*

*First of all, [AI] should be fast. There is always time pressure.*

*If I could see 30 patients in a day, and actually close out my charts by 6pm, smiling, and get home for dinner, I’d be happy.*

*At the moment we use a system called MedicalDirector [143] where we need to type in most of the notes. If you’re talking about an actual language processing system, where when we are talking it starts to record and then translate the consultation into clinical notes, I think that will be useful, If there were a program that could record my notes without me needing to sit and do it afterwards, that would save me time.*

*It’d do it even faster.*

*I think it would save time from googling. You know, I use the symptom calculators, I don’t know what they’re called, and you’ll enter your symptoms and see if it . . . what it might add up to, to get a little bit deeper, I think that would maybe give a little more detail.*

*In terms of note-taking I wasn’t sure when I sign up for your study, when you meant auto texting. So, I know like when I try to email on Gmail, they recognise what sentence I might want to write and give you possible text. So even that will speed up the process.*

*I suppose in an ideal world, I would have minimal hands-on input into the computer. But it would record my interactions and perform the functions that I need to function seamlessly, quickly, accurately.*

*It would be great if you could go, hey Siri, can you print off this radiology X-ray.*

*While a radiologist might manage 60 diagnostic findings a day, the AI could work day and night and deliver perhaps 180 or 200 findings. And if that happens with similar quality, then [...] you could examine many more patients than a human alone could.*

##### Skepticism about AI in primary care [20,22,24,27–29,31]

*Right now I don’t know anyone that’s...really using an AI tool beyond some gimmick… I don’t really know anyone that’s using something very sensible… we just tend to use apps on our phone that are just very convenient point of reference tools…*

*Is it really the physicians that should deal with this in the first place?*

*I’ve really come to realize that we have the ability with these systems because they can look at things that human cognition can’t to really spot or identify things where people’s equity is being compromised, either intentionally or unintentionally, and hopefully create better systems for people to live in. And here comes the half-empty side of me, I don’t think that’s going to happen. I really just don’t.*

*I feel like right when I get efficient, something new gets introduced.*

*Maybe it may show up too much information too if it’s not able to filter out what is important, not important, it may show a huge list of problems there, which is based on symptoms rather than diagnosis. So that could be a problem, too.*

*The system is all about the patient’s satisfaction. Is there any of that focus on physician satisfaction?*

*There’s a lot of conversation that you don’t necessarily need in the notes, so it’s got to be selective, doesn’t it?*

*When anyone writes their notes . . . there’s a degree of subjectivity in there. As a clinician, I’m really deciding what I feel are the key points that are worthwhile to document. I question how well a computer program could identify the same key points that I identify.*

*The other issue is the inability of a machine/AI to be able to skillfully ascertain the data required from a patient for correct analysis.*

*Transcription as in verbatim transcription will not be very helpful because it’s just too cumbersome [. . . ] But if there was a technology that can get the key information out of a conversation.*

*I can see potential for distraction, but I can also see potential for glossing over of information. For example, if I got a patient to free input a whole lot of data before I saw them, and they said no to some questions and yes to others, then I probably would be more inclined not to ask them the exact same questions again and be focused more perhaps on the stuff they’ve said yes to.*

*[...] in routine cases, [AI] would not be a time saver for me.*

*If I actually spend 10 minutes with a patient, right, in an ideal world this [the AI assistant in the video] will compute everything … We have what the patient needs, so I'm quickly looking, yeah, that's about right, yeah, save. That's it. That's an ideal outcome. In reality, what will happen is, no, no, no, delete, delete, delete.*

*oftentimes, you have to adapt your workflow and sometimes your clinical processes to accommodate the tool where I really strongly feel it needs to be the reverse*

*My concern is that like everything else that we have tried to do to make things better in medicine is that it actually makes things harder on the physician and creates more work for us instead of less work.*

*It’s like having a student with me all the time, where I’ve got to just double check everything.*

*We are going to add two extra patients per session because now we have help there. So unfortunately, sometimes more help is used in a negative way.*

### *Theme 3: Data are the cornerstone of AI development*

##### Privacy, safety, and confidence [20-24,26-32].

*This is indispensable, so if you want to develop artificial intelligence in the medical context, you cannot do it without patient data.*

*Both developing AI to help the GP and cure illnesses are in the same category. They are both equally good causes that I would share my data for.*

*Patient data are very sensitive data. Disease data are very sensitive data. [There is the risk that] they are passed on somewhere, that some authorities who have nothing to do with it or should have nothing to do with it could intercept the data and use this to the disadvantage of the patients.*

*I think it’s really important to clearly set out what the data be used for and ensure that the patient understands how the confidential confidentiality and privacy still applies in those situations.*

*...it must be ensured that data is deidentified...Overall, the process must be transparent, you must always be able to understand what is done with the data and how it is processed. Exactly...who processes the data, what research projects are being carried out...*

*Part of it would be an explanation of how it works from a privacy level. What is listened by the computer during a consult? How that information is stored. Is it stored locally? Does it get sent to the Cloud? Is it processed in the Cloud, or is it processed on a local machine? And how secure is that? Is it encrypted?.*

*...I would have a strange feeling if I knew data from various practices were being tapped and I had no idea what was happening with them. So, I think that would be fundamentally the wrong way to go about it.*

*My medical record is really boring, so my personal information would probably be more critical to share, although I share them the most already.*

*There has to be some sort of limit, so everyone cannot snoop around.*

*I have a sleep-app, that measures when I go to bed and when I wake up, and sometimes you can go to bed late and I have a sleeping illness, and I think what if the job center discovered that… What can they use it for? It would probably not benefit me.*

*I’m not sure about confidentiality, whether anyone who looks at the notes needs to know everything that is going on or not. I’m not sure how we can manage that. Though, I don’t think that will be a problem because I think once the patient is coming to see you, it means that you have access to the whole set of notes.*

*It’s understandable because it’s people’s medical information.*

*Of course, it is also important to me that there is corresponding data security. I do not want the patients and us to be completely transparent. That is certainly not in the overall interest.*

*It's always the problem of hacking, and somebody can know what exactly you do and can track the patient record.*

*...and same with the privacy concerns, there are already data leaks happening all the time and I think with more and more patient data being stored on an AI system or a computer system, it’ll just be even more important to safeguard that data.*

*I was just gonna say another concern that I think I would have, just because of the way our world is evolving and revolving, is can that artificial intelligence be hacked? Who can control that? …I don’t know. Because any time you have a computerized program, I don’t care what anybody says, it can and it will get hacked because there’s always somebody that’s out there just to do evil rather than good.*

*I think the biggest challenge as I’m sure you are well aware of, is around data standardization. The actual existence of that data, the interoperability of that data, the ability for that data to be machine interpretable.*

*…deployment of the algorithm, would it have led to better outcomes? And, importantly, did it present any risks?... – all clinicians are socialized into this ethic of, “The most important thing is that I protect my patients.” And so, that mentality, it pervades the healthcare professional culture, and especially medicine.*

*…as we move into these AI platforms, the concern I have is a lot of the algorithms are proprietary and [people] were very secretive in how they’re organizing these - what’s on the back end and how trusting can I be of what it’s telling me?*

*If there’s no RCT [randomized control trial] then I don’t know that it’s safe, and I don’t know that it’s effective, and I’m not going to deploy it in relation to my clinical decision making. Maybe practice management is different, but [for] any kind of clinical intervention that would involve AI, the lack of a traditional evidence base on it presents major challenges to moving forward.*

*AI tools. It’s gonna do what it’s gonna do. I think that I look forward with excitement… but I agree that there’s definitely some flaws, and there definitely needs to be some markers in there, at least right now, that can also protect people. But it’s going to be positive if we can get those safe markers in.*

*I believe the doctor always has the responsibility to be checking for you, and you’re his responsibility, you know? The AI is not responsible; that’s just a tool.*

*I think that part of what can help physicians [manage] our medical legal liability and risk is education and training around what these technologies are and what their purpose is…It's going to take a lot of awareness building among physicians to stay on top of how we can practice safely with the best interests of our patients in mind as these technologies become more widespread.*

*You could imagine a nightmare scenario where something bad happens … they subpoena the records or something, and you've got years of consultation. These consultations are stored in your records, and they run search terms. A person maybe had a cancer or something, so they run through 20 symptoms of their cancer through the whole thing, and then identify every instance that was mentioned … and they say, “all of these were mentioned—how come you missed it?” Or something like that … I mean, in a nutshell … patient safety is good—it's prime, but the doctor's safety should be there [too].*

*I think that’s always something that as providers that’s bred into you, to be concerned about liability. To me right now I think of the EMR as ammunition for lots of things, like in the past we didn’t have that, it was their word against ours, but now everything’s trackable.*

*If the system is saying, ‘Hey, this person has severe sleep apnea’, and what if they get in a car accident tomorrow and we had that data today?*

*I spend a lot of time going through refilling prescriptions… just looking at, “Oh, there’s John, his blood pressure’s good …three more months or a years’ supply.” Similar for maybe diabetic refills…. could, in the back end, the AI read maybe an NLP [natural language processing], to look at those indicators of blood pressure, lab parameters, and have some intuition, this would be fine, to release that. …What would be the medical legal implications of that, obviously?*

*If everything was completely recorded and stored, [. . . ] the entire conversation, if that was recorded and stored, and the doctor missed something, then that could be used against the doctor. That the patient mentioned it, but it wasn’t listed or written, or a doctor accidentally missed that detail. Then that could be a problem.*

*Technology will be supporting clinicians in the very near future – the issue is responsibility and liability in legal terms for such tools.*

##### Corporate responsibility [20,22,23,25,29]

*The problem is that large companies use AI to gain access to lucrative patients and to control them via AI.*

*the other part is [what is] behind the – maybe the dirty side of AI, which is the monetization of big data.*

*how this data is being used and whether there were any concerns about this data being used by a [private] company.*

*[what] we need to add is a way to make sure that personal health information is protected and that we don't step outside social licence.*

*...how can we make sure that the companies don’t just profit from it, and how do we make sure that people aren’t disadvantaged, and do people really understand that their data are being used.*

*…the business model behind these technologies is where I think the most important ethics conversation is to be had at the moment.*

*It's the big conglomerates that want to use this and I know they use it in other areas like social studies. But health data, people feel discomfort, right.*

*Where is the investment going to come from? People are going to be skeptical about investments into that, when our health care system is already, from the public’s perception, really struggling.*

*Like who decides what’s good or bad? It’s relative depending on whatever company wants to make a bunch of money off their data. That’s what I’m the most nervous is about the corporate side of it. Who is regulating it? Who is saying this algorithm is good to go? There’s no . . . there isn’t that yet.*

##### Bias and mistakes [20-26,28,29,32]

*The AI will recommend examinations that I would personally put last, ie. it will possibly lead to so-called device medicine, involving a lot of safeguard diagnostics, which I consider to be quite questionable.*

*we want to make sure that the tools we use don’t create new problems… they are an opportunity to address some of the biases that already exist in our system. . .and that these tools are probably only as good as the data we provide them.*

*…maybe it’s [AI] giving me information based on… the data fed into it if there was an uptick on aortic stenosis or rheumatoid valve disease, etcetera and it’s erroneously cautioning me or giving me a flag that you really wouldn’t worry about otherwise.*

*AI is not a living creature and depending on what you feed it with, it can learn different things, so it is important to be critical.*

*Now, something that could come into place and relieve some of that burden, that’s great. But, if it could add to that stress at all, what if the AI gets it wrong?*

*The thing I’m apprehensive about is, how are we teaching AI these things because some of those biases could leak in.*

*My concerns around AI in medicine have most to do with the space of accuracy. And a tool that I feel is reliable.*

*I think another big challenge with AI is the sort of algorithmic bias that can come with the development of the AI systems, another challenge to think about [is] the actual development of the AI that’s being used and ensuring that it’s not biased and it’s really promoting an equitable system.*

*I think the risks would be if somebody inputted something wrong and it then prompting you to do something. Like the data at the end is only as good as how you input the data at the beginning.*

*One thing . . . I’m not sure if this is exactly what you’re asking, but just a thought, prejudices that people can have, like it could absorb those or it could be taught to work against them, like a lot of people who are overweight have said that their providers assume that that’s the cause and ignore doing other tests or pursuing other avenues, and if an AI wasn’t going to make the assumption that that was what was the problem, then that would be good, but if it was learning from people around it that it should make that assumption, then it would perpetuate the problem.*

*There’s a lot of discrepancies in the medical record I must say, especially now that you can see your portal. I know I’ve seen things saying that certain things were done or about myself and procedures that were totally not true. So I’ve had a lot of different things in my medical chart that are inaccurate, very inaccurate, so if they’re training an artificial intelligence that this is facts, it’s like, well no.*

*Prejudices that people can have, like it could absorb those or it could be taught to work against them, like a lot of people who are overweight have said that their providers assume that that’s the cause and ignore doing other tests or pursuing other avenues, and if an AI wasn’t going to make the assumption that that was what was the problem, then that would be good, but if it was learning from people around it that it should make that assumption, then it would perpetuate the problem.*

*I have some background in electronics, and one thing you can guarantee with electronics is they will fail. Might not be now, might never happen in 10, 20 years. The way things are made, ‘cause I’ve actually worked in the industry of making medical equipment, it’s all about using the cheapest method to get the end result. Well, electronics fail. They just do.*

*If, for example, they wrote schizophrenia instead of schizotypal personality disorder. Somebody then has a diagnosis that maybe was incorrect because somebody clicked on the wrong button. Then everybody else, the GP, then the next GP and then the other mental health service might then label this person with a condition that they don’t have.*

*Perhaps a medication error, somebody makes a mistake on the discharge summary about the medication that was dispensed on discharge [. . . ] if you’re doing it manually, might think that dose doesn’t sound correct. Whereas if it’s done automatically the GP might not think about what’s been copied and so the error then gets multiplied.*

*And also, in case it recognizes the discharge summary. I guess there’s a lot of difference between the patient does not have a penicillin allergy versus the patient developed a penicillin allergy.*

*When It [text automation system] emphasises things, sometimes it might emphasise one part and then the doctor automatically doesn’t look at the other parts. [...] Then you might get an issue where the doctor using the summarised documents, actions only part of the recommendations from the hospital.*

*It might misrecognise a mistyped medication. Instead of, say, prednisone, it takes prednisolone. It’s a minor error. But you know what I mean, it accidentally puts in the wrong medication because of, it could be the errors in someone else’s letter.*

*Men and women are quite different, in terms of their presentations of diseases, and if it's [the AI assistant in the video] learning on the basis of a male—a man's idea of a disease, and if I do not follow that, and something does happen, who gets sued in that intervention, if I chose to ignore that bit of information?*

*fall through the cracks.*

*… A lot of the input data coming so far is from white males—so a lot of the algorithms and all of the learning at the moment is largely based on white male thinking … so there are inherent biases already within their programs in the way that [data is being collected]—even the idea of drawing conclusions about the diagnosis.*

*I guess I’m kinda curious as to wonder how would people with other challenges use that because I live in a boarding lodge, and there’s three or four people that live there that have been there for umpteen years, and the care there is not adequate for them. They were placed there after our state hospital closed, but there’s no way they would be able to understand and communicate a chat box. So I’m just wondering ‘cause you don’t wanna . . . I guess I’d be afraid that they weren’t gonna be helped or get skipped over.*

*And as we become more and more diverse, and even our genealogies become more and more diverse, you want as much data as possible from as many different backgrounds as possible. And I would highly question if all of the data points were coming from one set. I think I’ve also heard statistics where like a lot of studies don’t include women. They are starting to include more women, but a lot of the original studies on medications involved only included men, and so there hasn’t been a lot of data on how it affects women’s bodies. So I would question heavily if it wasn’t more diverse.*

### *Theme 4: Barriers and facilitators to AI in primary care*

##### Social factors influence perceptions [20,24-27,29-32]

*I am convinced it needs much work because there is certainly much resistance, which clearly depends on age.*

*Unless you’re like my uncle, my uncle says that we’d all be better off if we went back to the times where all this technology hadn’t been invented and computers hadn’t been invented. He says computers are a fad.*

*I can’t see there being a barrier implementing it into our workplace because I think for the most part our providers are almost eager for this type of information. Anything that can help them and help their client.*

*I also know young colleagues who are my age, and they also have strong reservations [regarding AI].*

*For our generation, it is really uncomfortable. But young generations who have grown up with technology as this . . . well I look at my grandkids who are little, and that’s their world. So would they feel as uncomfortable as I might in this? I think, maybe not. Is it . . . are you more secure, do you feel better about what that computer is telling you than I would feel about it?*

*Doctors more towards near retirement, they might not be happy to take up the new technology. . . . it comes back o the medical-legal aspect.*

*I would really have to understand and have folks there that also understand how it works or can teach us. So we’re not just blindly relying on technology in a setting where we’re working with humans.*

*Well, there are also people in my generation who were already technically inclined [...]. So, I think that’s the key to why people [would use AI] or not.*

*We worked a little bit with some clinical tools that aid in decision-making when we transitioned [to our new] EMR, but I would say it was very user driven and not so much analytic power on the computing system.*

*The only barrier would be that we’ve had some bad experiences with EMRs and the functionality. So there might be a little bit of initial skepticism for something new.*

*A lot of us, any of us that have a smart phone are already using a form of AI. For example, voice to text. I use the Samsung version, and the more I use it, the more it can get my words accurately, and actually right now with my phone in the Samsung version, it gets it right I would say 95% of the time so…*

*Humans can’t win chess against Big Blue anymore because it has found ways not programmed in initially, and then there’s another… there’s a game that is an Asian game that is very, very popular, and it’s the same thing. The best grand masters of that can’t beat the machines because the artificial intelligence has figured out, so the humans have set template and then the artificial intelligence boom-boom-boom-boom.*

*I think 80% of that combined group would be onboard. 20% do not have the faith, are not mentally advanced with technology. There were a few people that transitioning to a new EMR was like cutting off an arm.*

*These days a lot of airplane travel is on autopilot that's done by computers, based on our inputs and everything else, but at the same time, would anyone here get into a flight that's entirely pilotless?*

*It sounds like it could be very positive. I don’t have too many reservations because I feel like we already store all of our data in an electronic format.*

*In essence, we’re providing a bunch of free care, which, you know, is not sustainable.*

*The people who will get it are the people who can pay for the compute. And so that’s my biggest fear is that we will leave out the poorest people from getting the best care.*

*I mean, … that information is wonderful, but who’s gonna get it after the doctors look at it is my big thing. Is the insurance company gonna take it, and now all of a sudden … my premium doubles for health insurance?*

*So it sounds expensive, and health care is already fairly expensive. To go on his note, a lot of times you can get something that works just as well for a lot less or you could get something super fancy, that makes you think, hey I got this big fancy thing, but it really doesn’t do any better than the original cheaper version.*

*Is insurance only gonna cover what the machine says it is and not look for anything else? There is no reason for further diagnostics because the machine already did it? I mean we already have a situation in our healthcare system where money comes into play for diagnosing things.*

*there could be bias in your software. Because these factors are not all equal, they have different weights of importance. And so how that’s applied will affect how good the results are going to be.*

##### Must-have factors [20,21,23,24,26-31]

*We need to develop machine learning that has the capability … to accommodate professional preferences and styles … It's adaptive and it supports you based on your particular consultation and decision-making processes.*

*I have a 15-year-old who's learning how to drive. We have one car that has sensors and all sorts of safety features, and another car that doesn't….I feel like he needs to understand how to operate a vehicle at its base level before he can really make use of [safety features]…Maybe we need to emphasize the diagnostic reasoning, the history and physical pieces and the test ordering first, and introduce AI to that senior clinical learner…rather than right off the bat, so that they've got those building blocks behind them.*

*I think … we're not asking the right question about the future role of technology in healing. Because the future is not better documenting disease … the future is in how we might empower people towards a better or subjective experience of their life … the question is what is the role of data in healing … the problem is you guys [technology designers/researchers] are conflating efficient data management with healing.*

*If you build the brain power, AI power to do something, it has to be really user-friendly at the end of the day.*

*That’s how frontline staff tend to look at data, does it make my life easier? Is it easier to look at? Does it give me the information that I want when I want it? Is it reliable? And if it’s not then I’m going to do it an easier way.*

*Especially as our patients are becoming more and more complicated, we need to ensure that the EMR supports us and does not make us work harder, so we can sink those efforts and energies into the patient.*

*I must know how [AI] obtains information and how [it] works.*

*So when this intelligence is built we have to test it, right? We have to test it to make sure that it’s helping correctly, and that to me represents a big challenge and one we don’t wanna jump into and see what happens. We’ve gotta be very careful there.*

*Doctor should have control over whether or not the whole conversation is recorded and stored. Or whether their conversation is recorded, stored, processed, and then deleted.*

*The question is whether they will be acceptable to patients although they may be very accessible compared to the current system.*

*The somewhat blunt tool of technology as it stands will need to evolve some way before the culture of clinicians and patients will accept it.*

*I suppose with physicians, evidence that something works won’t necessarily convince them to do it, but the lack of evidence about something will convince them not to do it.*

*I think it would have to be run as informative but not actionable for a while first where it just demonstrates that it is working, that it is effective before people would start to trust it.*

*At this point, I want to be able to get a logical explanation.*

*Well, I think that’s progress. I’m all for progress, and regardless what we think, some things are gonna go this way anyway, and it’s progress, and as it comes, sometimes, like I said, you gradually accept this… because I swore years ago I wasn’t going to have a computer. That was just ridiculous… but things change, because as things are changing, we accept it. But technology is here to stay, and there are gonna be things in another 50 years that we wouldn’t dream of and possibly dream of, so that’s where it’s going, and I think it will be accepted.*

*I feel like the future of AI just depends on how we choose to use it. The impact will be what we choose it to be. … Because it’s moldable, it’s not going to do anything that we don’t allow it to do.*

*[I]t’s important to take into account that people, depending on what the AI comes out with, people might not be willing to go with what that is, they might need alternates. And also just the question of creativity, like what if the solution were actually something where you would have to think outside the box? … What if it’s something they haven’t encountered before?*

*I think it all comes back to choice, though, I think everybody’s getting the mentality that, and maybe I’m wrong, but that an AI is being pushed, but at the end of the day, our choice is still our choice, and it’s not being taken away.*

*So I’d rather know what they’re observing and, if it’s [AI] wrong, I would [want to] be able to correct it rather than have them just collect data and make assumptions.*

*As long as I could give the final yes or no.*

*And just whatever the doctor approves to go into the EMR (Electronic Medical Record) is what remains.*

*I would participate only [on a] voluntarily [basis].*

*I wouldn’t use it blindly I guess, and so I’d be confident that it would save me time but not replace me thinking, which is not the aim, for me it’s the saving time.*

*You’d need to feel confident it was accurate, and that information was reliable, that it was able to properly look through, for example, if it’s a bunch of old notes and things, that it actually was checking everything accurately and extracting the right stuff.*

*I think that general practice obviously, in the medical field in general we’re quite behind with trying new technologies in clinical practice. Bear in mind that I probably have a different view compared to other GPs perhaps, because I am in the entrepreneurial and health tech start-up space. I’m not sure if all doctors would feel that way. And I come from a different demographic, obviously. A younger demographic, maybe, compared to the 50-year-old GPs out there.*

##### Designing with, rather than for [20,22,24]

*designing with, rather than for.*

*…Engaging people in the process of building tools would be super helpful so they actually see what’s going on and have some feedback and contribution to it.*

*So there’s a lot of trust and, you know, the actual programmers, most by and large don’t go to medical school themselves. There's a lot of interdisciplinary sort of interaction that has to occur, because when they're developing the models, the ethics that we adhere to as part of practicing clinicians, must be embedded into – right from the get-go, even before actually writing the code, in the conception of the model itself.*

*There’s a lot of oversight that needs to occur at that level, before it’s even deployed… Because what AI will do is, it will just spit out whatever you put in. You know, the data in, data out thing is never more true for AI, and the models need to be calibrated at that programmatic level to make sure that it’s not exacerbating existing sort of negative issues that are embedded in our healthcare system, you know.*

*Instead of just letting the cat out of the bag and seeing what happens, you want to make sure that everyone that is going to be interacting with it has accurate expectations and has been educated on what role this is supposed to play.*

*…that co-design piece of having the end users—so most likely nurses, doctors, nurse practitioners, anyone who’s going to be using the technology really needs to be involved in the development and co-design of the technology from the beginning. …right now, with other types of technology, it will be this tech company that’s developing this great system and they only consult the end users when it’s finished and then it’s almost too late to kind of incorporate things that really should’ve been included from the beginning. So that is something that would be really important to emphasise and could help with a successful implementation of any AI technology as well.*

*I think that they [patients and practitioners] [need to] know what goes into the black box. I’d want to make [sure] that it was actually explained and articulated how the end result came about. I just wouldn’t want any assumptions to be made. Sometimes at the system level decisions are made that may not take into consideration all the different aspects. I don’t think that would happen but having all of the different pieces put together is important, the contextual pieces.*

**References**

20. Darcel K, Upshaw T, Craig-Neil A, et al. Implementing artificial intelligence in Canadian primary care: Barriers and strategies identified through a national deliberative dialogue. Mohammadzadeh A, ed. PLoS One 2023;18(2):e0281733. doi:10.1371/journal.pone.0281733

21. Upshaw TL, Craig-Neil A, Macklin J, et al. Priorities for artificial intelligence applications in primary care: a Canadian deliberative dialogue with patients, providers, and health system leaders. J Am Board Fam Med 2023;36(2):210-220. doi:10.3122/jabfm.2022.220171R1

22. Terry AL, Kueper JK, Beleno R, et al. Is primary health care ready for artificial intelligence? What do primary health care stakeholders say? BMC Med Inform Decis Mak 2022;22(1):237. doi:10.1186/s12911-022-01984-6

23. Nash DM, Thorpe C, Brown JB, et al. Perceptions of artificial intelligence use in primary care: a qualitative study with providers and staff of Ontario community health centres. J Am Board Fam Med 2023;36(2):221-228. doi:10.3122/jabfm.2022.220177R2

24. Allen MR, Webb S, Mandvi A, Frieden M, Tai-Seale M, Kallenberg G. Navigating the doctor-patient-AI relationship - a mixed-methods study of physician attitudes toward artificial intelligence in primary care. BMC Prim Care 2024;25(1):42. doi:10.1186/s12875-024-02282-y

25. Richardson JP, Curtis S, Smith C, et al. A framework for examining patient attitudes regarding applications of artificial intelligence in healthcare. Digit Health 2022;8:205520762210890. doi:10.1177/20552076221089084

26. Richardson JP, Smith C, Curtis S, et al. Patient apprehensions about the use of artificial intelligence in healthcare. NPJ Digit Med 2021;4(1):140. doi:10.1038/s41746-021-00509-1

27. Kocaballi AB, Ijaz K, Laranjo L, et al. Envisioning an artificial intelligence documentation assistant for future primary care consultations: A co-design study with general practitioners.  J Am Med Inform Assoc 2020;27(11):1695-1704. doi:10.1093/jamia/ocaa131

28. Fraile Navarro D, Kocaballi AB, Dras M, Berkovsky S. Collaboration, not confrontation: understanding general practitioners’ attitudes towards natural language and text automation in clinical practice. ACM Trans Comput-Hum Interact 2023;30(2):1-34. doi:10.1145/3569893

29. Buck C, Doctor E, Hennrich J, Jöhnk J, Eymann T. General practitioners' attitudes toward artificial intelligence-enabled systems: Interview study. J Med Internet Res 2022;24(1):e28916. doi:10.2196/28916

30. Kamradt M, Poß-Doering R, Szecsenyi J. Exploring physician perspectives on using real-world care data for the development of artificial intelligence–based technologies in health care: qualitative study. JMIR Form Res 2022;6(5):e35367. doi:10.2196/35367

31. Blease C, Kaptchuk TJ, Bernstein MH, Mandl KD, Halamka JD, DesRoches CM. Artificial intelligence and the future of primary care: Exploratory qualitative study of UK general practitioners’ views. J Med Internet Res 2019;21(3):e12802. doi:10.2196/12802

32. Mikkelsen JG, Sørensen NL, Merrild CH, Jensen MB, Thomsen JL. Patient perspectives on data sharing regarding implementing and using artificial intelligence in general practice – a qualitative study. BMC Health Serv Res 2023;23(1):335. doi:10.1186/s12913-023-09324-8
