# Supplementary material for: Perceptions of, Barriers to, and Facilitators of the Use of AI in Primary Care: Systematic Review of Qualitative Studies
Source: J Med Internet Res. 2025 Jun 25;27:e71186. doi: 10.2196/71186 (PMC12242059; doi:10.2196/71186)
Supplement: Multimedia Appendix 5 [file jmir_v27i1e71186_app5.docx]

**Evidence Profile Table, GRADE CERQual**

| **Summarised review finding** | **Methodological limitations** | **Coherence** | **Adequacy** | **Relevance** | **GRADE-CERQual assessment of confidence** | **Ref**^a^ |
| --- | --- | --- | --- | --- | --- | --- |
| 1. **Change in the physician-patient relationship** | | | | | | |
| AI lacks the human-specific qualities essential for patient care, such as empathy and understanding, raising concerns about its impact on the physician-patient relationship and trust. Physicians also fear being reduced to data collectors and patients devaluing their clinical advice, potentially affecting job security | Minor concerns  **Explanation:** Three studies with minor [32, 33, 39] and one study with moderate methodological limitations [38] (primarily due to the limited approach for gathering information through open-ended survey questions). | No/Very minor concerns  **Explanation:** All the included articles present a consistent and aligned perspective. | No/Very minor concerns  **Explanation:** A large amount and depth of qualitative data. Twelve studies that collectively provided very rich information. | No/Very minor concerns  **Explanation:** Studies that provide important insights into the physician-patient relationship, involving employees, patients and students. | High confidence  **Explanation:** Minor concerns regarding methodological limitations (one study [38] using open ended survey questions). This study does not change the common ideas of the final conclusions on the quotations of the participants. No/Very minor concerns regarding coherence, No/Very minor concerns regarding adequacy, and No/Very minor concerns regarding relevance. | [20-29, 31-32] |

| **Summarised review finding** | **Methodological limitations** | **Coherence** | **Adequacy** | **Relevance** | **GRADE-CERQual assessment of confidence** | **Ref**^a^ |
| --- | --- | --- | --- | --- | --- | --- |
| 1. **AI as a partner for efficient time and information management** | | | | | | |
| The integration of AI in healthcare is expected to transform the field by improving data processing, diagnostic accuracy, and clinical decision-making. While some anticipate reduced workloads and increased patient interaction for caregivers, others worry that AI systems may not perform as expected, potentially increasing the overall burden of care. | Minor concerns  **Explanation**: Three studies had [32, 33, 39] methodological limitations, while one study [38] had moderate limitations (primarily due to the limited approach for gathering information through open-ended survey questions). | No/Very minor concerns  **Explanation**: All articles provided clear and uninterpreted information about possible future applications for the improvement of primary care development. Additionally, half of them also offered divergent perspectives concerning the potential complications of implementing IA. | No/Very minor concerns  **Explanation**: Twelve studies contributed a wealth of detailed and comprehensive data. | No/Very minor concerns  **Explanation**: The section presents relevant information on the perceptions of potential uses of IA and its future implications. | High confidence  **Explanation**: Minor concerns regarding methodological limitations (one study [38] using open ended survey questions). This study does not change the common ideas of the final conclusions on the quotations of the participants. No/Very minor concerns regarding coherence, No/Very minor concerns regarding adequacy, and No/Very minor concerns regarding relevance. | [20-25, 27-32] |

| **Summarised review finding** | **Methodological limitations** | **Coherence** | **Adequacy** | **Relevance** | **GRADE-CERQual assessment of confidence** | **Ref**^a^ |
| --- | --- | --- | --- | --- | --- | --- |
| 1. **Data are the cornerstone of AI development** | | | | | | |
| Participants raised concerns about the ethical and practical implications of integrating AI into healthcare, highlighting issues such as privacy violations, patient safety, data transparency, and biases in AI systems. There were also worries about the impact on medical autonomy, with constant data monitoring potentially limiting clinical decision-making. | Minor concerns  **Explanation**: Three studies with minor [32, 33, 39] methodological limitations. A single study [38] presented moderate methodological limitations (primarily due to the limited approach for gathering information through open-ended survey questions). | No/Very minor concerns  **Explanation**: The review presented information on the potential ethical and moral challenges associated with the implementation of IA without any interpretative input. | No/Very minor concerns  **Explanation**: Thirteen studies that collectively provided very rich data. | No/Very minor concerns  **Explanation**: This section presented a series of conclusions pertinent to the prospective deployment of artificial intelligence in the context of primary care | High confidence  **Explanation**: Minor concerns regarding methodological limitations (one study [38] using open ended survey questions). This study does not change the common ideas of the final conclusions on the quotations of the participants. No/Very minor concerns regarding coherence, No/Very minor concerns regarding adequacy, and No/Very minor concerns regarding relevance. | [20-32] |

| **Summarised review finding** | **Methodological limitations** | **Coherence** | **Adequacy** | **Relevance** | **GRADE-CERQual assessment of confidence** | **Ref**^a^ |
| --- | --- | --- | --- | --- | --- | --- |
| 1. **Barriers and facilitators to AI in primary care** | | | | | | |
| The social context influences how patients view their role in a technology-driven primary care system. Successful AI implementation in healthcare requires adaptability to professional preferences and a co-design approach involving healthcare professionals and patients to ensure tools meet clinical needs, promote transparency, and ease adoption. | Minor concerns  **Explanation**: Three studies with minor [32, 33, 39] and one study [38] with moderate methodological limitations (primarily due to the limited approach for gathering information through open-ended survey questions). | Moderate concerns  **Explanation**: Exists a moderate concern about coherence due to the way participants' ideas are interpreted in order to create this set of ideas. | No/Very minor concerns  **Explanation**: Thirteen studies that collectively provided very rich data. | No/Very minor concerns  **Explanation**: The review identified key factors influencing the adoption of IA in primary care. | High confidence  **Explanation**: Minor concerns regarding methodological limitations (one study [38] using open ended survey questions). This study does not change the common ideas of the final conclusions on the quotations of the participants. No/Very minor concerns regarding coherence, No/Very minor concerns regarding adequacy, and No/Very minor concerns regarding relevance. | [20-32] |

^a^ References

**References**

20. Darcel K, Upshaw T, Craig-Neil A, et al. Implementing artificial intelligence in Canadian primary care: Barriers and strategies identified through a national deliberative dialogue. Mohammadzadeh A, ed. PLoS One 2023;18(2):e0281733. doi:10.1371/journal.pone.0281733

21. Upshaw TL, Craig-Neil A, Macklin J, et al. Priorities for artificial intelligence applications in primary care: a Canadian deliberative dialogue with patients, providers, and health system leaders. J Am Board Fam Med 2023;36(2):210-220. doi:10.3122/jabfm.2022.220171R1

22. Terry AL, Kueper JK, Beleno R, et al. Is primary health care ready for artificial intelligence? What do primary health care stakeholders say? BMC Med Inform Decis Mak 2022;22(1):237. doi:10.1186/s12911-022-01984-6

23. Nash DM, Thorpe C, Brown JB, et al. Perceptions of artificial intelligence use in primary care: a qualitative study with providers and staff of Ontario community health centres. J Am Board Fam Med 2023;36(2):221-228. doi:10.3122/jabfm.2022.220177R2

24. Allen MR, Webb S, Mandvi A, Frieden M, Tai-Seale M, Kallenberg G. Navigating the doctor-patient-AI relationship - a mixed-methods study of physician attitudes toward artificial intelligence in primary care. BMC Prim Care 2024;25(1):42. doi:10.1186/s12875-024-02282-y

25. Richardson JP, Curtis S, Smith C, et al. A framework for examining patient attitudes regarding applications of artificial intelligence in healthcare. Digit Health 2022;8:205520762210890. doi:10.1177/20552076221089084

26. Richardson JP, Smith C, Curtis S, et al. Patient apprehensions about the use of artificial intelligence in healthcare. NPJ Digit Med 2021;4(1):140. doi:10.1038/s41746-021-00509-1

27. Kocaballi AB, Ijaz K, Laranjo L, et al. Envisioning an artificial intelligence documentation assistant for future primary care consultations: A co-design study with general practitioners.  J Am Med Inform Assoc 2020;27(11):1695-1704. doi:10.1093/jamia/ocaa131

28. Fraile Navarro D, Kocaballi AB, Dras M, Berkovsky S. Collaboration, not confrontation: understanding general practitioners’ attitudes towards natural language and text automation in clinical practice. ACM Trans Comput-Hum Interact 2023;30(2):1-34. doi:10.1145/3569893

29. Buck C, Doctor E, Hennrich J, Jöhnk J, Eymann T. General practitioners' attitudes toward artificial intelligence-enabled systems: Interview study. J Med Internet Res 2022;24(1):e28916. doi:10.2196/28916

30. Kamradt M, Poß-Doering R, Szecsenyi J. Exploring physician perspectives on using real-world care data for the development of artificial intelligence–based technologies in health care: qualitative study. JMIR Form Res 2022;6(5):e35367. doi:10.2196/35367

31. Blease C, Kaptchuk TJ, Bernstein MH, Mandl KD, Halamka JD, DesRoches CM. Artificial intelligence and the future of primary care: Exploratory qualitative study of UK general practitioners’ views. J Med Internet Res 2019;21(3):e12802. doi:10.2196/12802

32. Mikkelsen JG, Sørensen NL, Merrild CH, Jensen MB, Thomsen JL. Patient perspectives on data sharing regarding implementing and using artificial intelligence in general practice – a qualitative study. BMC Health Serv Res 2023;23(1):335. doi:10.1186/s12913-023-09324-8
